# Miniaturizing VEGF: Peptides mimicking the discontinuous VEGF receptor-binding site modulate the angiogenic response

**DOI:** 10.1038/srep31295

**Published:** 2016-08-08

**Authors:** Lucia De Rosa, Federica Finetti, Donatella Diana, Rossella Di Stasi, Sara Auriemma, Alessandra Romanelli, Roberto Fattorusso, Marina Ziche, Lucia Morbidelli, Luca Domenico D’Andrea

**Affiliations:** 1 Istituto di Biostrutture e Bioimmagini, CNR, Via Mezzocannone 16, 80134, Napoli, Italy; 2 Dipartimento di Scienze della Vita, Università di Siena, Via A. Moro 2, 53100 Siena, Italy; 3 Dipartimento di Farmacia, Università di Napoli “Federico II”, via Mezzocannone 16, 80134, Napoli, Italy; 4 Dipartimento di Scienze e Tecnologie Ambientali, Biologiche e Farmaceutiche, Seconda Università di Napoli, via A. Vivaldi 43, 81100, Caserta, Italy

## Abstract

The angiogenic properties of VEGF are mediated through the binding of VEGF to its receptor VEGFR2. The VEGF/VEGFR interface is constituted by a discontinuous binding region distributed on both VEGF monomers. We attempted to reproduce this discontinuous binding site by covalently linking into a single molecular entity two VEGF segments involved in receptor recognition. We designed and synthesized by chemical ligation a set of peptides differing in length and flexibility of the molecular linker joining the two VEGF segments. The biological activity of the peptides was characterized *in vitro* and *in vivo* showing a VEGF-like activity. The most biologically active mini-VEGF was further analyzed by NMR to determine the atomic details of its interaction with the receptor.

All biological processes are finely regulated by a network of interactions between proteins whose characterization at molecular level can promote the design of novel protein binder drugs with therapeutic and diagnostic applications. Peptides and peptidomimetics have been widely explored as protein-protein interaction modulators as they are advantageous with respect to protein-based (including antibodies) molecules and small organic compounds in spite of pharmacokinetic limitations^1^. In the last years, several examples of protein-binder peptides have been reported, achieved using either structure-based design, combinatorial or computational approaches. In particular, considering that molecular recognition between proteins is often mediated by surface exposed loop or secondary structure motifs such as β-hairpins and α-helices, many efforts have been devoted to the development of peptides reproducing these protein interface elements^1^,^2^. Less explored and more challenging is the design of protein interface mimetic peptides reproducing multiple and discontinuous binding sites. A discontinuous binding site is constituted by peptide segments which are close in the protein three-dimensional structure but distant in the primary sequence. Examples of discontinuous protein binding site/epitopes mimicry using peptides have been reported. They have been assembled by fusing into a single molecular entity two or more linear amino acid segments using molecular scaffolds or linkers^3^,^4^,^5^,^6^,^7^,^8^,^9^,^10^,^11^,^12^,^13^ or selected by phage display from libraries of mimotopes^14^. We explored the design of peptides mimicking two interacting regions of the Vascular Endothelial Growth Factor (VEGF) for its receptor.

VEGF is the main regulator of angiogenesis, a fundamental process for healing, reproduction and embryonic development^15^. The design of novel and effective angiogenic modulators is eliciting a considerable interest for therapeutic^16^,^17^ and diagnostic applications^18^. VEGF is a homodimeric glycoprotein, covalently bound by two disulfide bonds, characterized by a cystine knot motif^19^. It binds to two receptors on the surface of endothelial cells (ECs) (VEGFR1 and VEGFR2). The binding of VEGF to its receptors induces receptor dimerization and phosphorylation of the intracellular kinase domain which activate the intracellular pathways ending in EC proliferation and migration^20^. The analysis of the x-ray crystal structure of the complex between VEGF and the domain 2 of VEGFR1 (VEGFR1D2) (1flt.pdb)^21^ shows that VEGF interaction interface is defined by a discontinuous surface comprising binding residues distributed in three regions belonging to both VEGF monomers: the N-terminal helix (residues 17–25), the loop joining strand β3 and β4 (residues 61–66) of one VEGF monomer, and the β-hairpin encompassing strand β5 and β6 (residues 79–93) of the other VEGF monomer (Supplementary Fig. S1). We already showed that conformational constrained peptides reproducing the α-helix or the β-hairpin VEGF regions are able to bind to the VEGF receptors and modulate VEGF biological response^22^,^23^,^24^,^25^. In particular, either VEGFR antagonist^22^,^24^ and agonist^23^,^25^ were found.

In this work, we report the design of a set of peptides mimicking two secondary structure elements of VEGF (α-helix 17–25 and β-hairpin 79–93) involved in receptor recognition. The two linear amino acid segments were synthesized by solid-phase synthesis and conjugated by a chemical ligation reaction through amino acid spacers of variable length and flexibility. Their biological activity has been investigated by *in vitro* and *in vivo* assays, highlighting a VEGF-like biological activity. The structural features of the most biologically effective peptide interacting with the recombinant second domain of VEGFR1 were investigated by NMR techniques.

## Results and Discussion

### Peptides design

**Figure 1:**
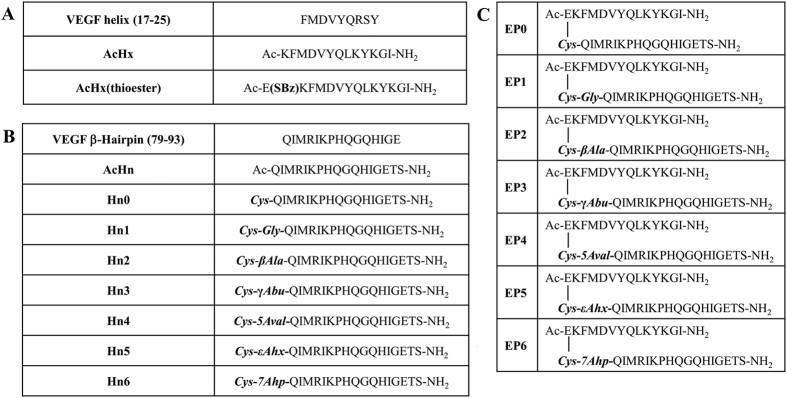
Amino acid sequences and nomenclature of peptides. (**A**) Peptides (Hx) derived from the VEGF helix (17-25). Hx peptide was acetylated at the N-terminus and amidated at the C-terminus. Hx was functionalized with a benzylthioester group on the side chain of Glu1. (**B**) Peptides (Hn) derived from the VEGF β-hairpin (79-93). Hn peptides differ for the length of the spacer. Hn peptides were amidated at the C-terminus. (**C**) Peptides obtained from the chemical ligation reaction between AcHx-thioester and the Hn peptides. EP number refers to the Hn peptide used. The chemical ligation reaction resulted in the formation of an isopeptide bond linking the carboxylic side chain of Hx Glu1 with Hn cysteine α-amino group. βAla = β-alanine; γAbu = γ-aminobutyric acid; 5Aval = 5-aminovaleric acid; εAhx = ε-aminocaproic acid; 7Ahp = 7- aminoheptanoic acid.

The analysis of the x-ray crystal structure of the complex between VEGF-VEGFR1D2 (1flt.pdb)^21^ shows that the N-terminal helix 17–25 and the β-hairpin fragments 79–93, belonging to two different monomers of VEGF, present their N-terminal extremities in close proximity (Supplementary Fig. S1). In particular, Val15 and Met78 are the closest residues. The VEGF segments 15–25 and 79–93 were chosen as target sequences to be covalently linked into a single molecular entity. The N-terminal helix, including five VEGF binding residues, was modified at the C-terminal end introducing a C-capping region^26^ in analogy with our previous design^22^,^23^. Val15 was replaced with a glutamic residue for synthetic reason. The N- and C-termini were acetylated and amidated respectively to stabilize the helical conformation^27^,^28^. The β-hairpin fragment 79–93 was extended at the C-terminus adding the dipeptide Thr-Ser to make the two strands approximately of the same length. Threonine and serine were selected as they represent a good compromise between intrinsic β-sheet forming propensity and solubility^29^. Finally, the C-terminus was amidated to improve peptide stability. The two peptide fragments Hx (Helix) and Hn (Hairpin), which are related but not identical to our previously described peptide^22^,^23^,^24^,^25^, were covalently linked by a spacer joining Glu side chain located at the N-terminus of Hx and the N-terminal amino group of Hn. We designed a spacer with different size and flexibility. The spacer is composed of a Cys, to allow the chemical ligation with the γ-thioester of Glu in Hx peptide, and an amino acid residue with a different number of backbone atoms (none, glycine, β-alanine, γ-aminobutyric acid, 5-aminovaleric acid, ε-aminocaproic acid and 7-aminoheptanoic acid). Peptide sequences are reported in Fig. 1.

### Peptide synthesis

The precursor Hx and Hn peptides were prepared by solid phase using Fmoc chemistry^30^. Hx and Hn peptides were respectively prepared as thioester and cysteinyl derivatives (Supplementary Fig. S2). These two functional groups were introduced in order to link Hx and Hn by a chemical ligation reaction^31^.

The N-terminal Glu residue of Hx peptide was thioesterified in solid phase after selective deprotection of the side chain carboxy group from the O-Allyl protecting group using tetrakis(triphenylphosphine)palladium(0) (Pd(PPh_3_)_4_) and phenylsilane^32^. Resin cleavage and HPLC purification afforded the pure deprotected AcHx(thioester) peptide which was purified and characterized by LC-MS (Supplementary Table S1). Seven variants of Hn peptide (namely Hn0 to Hn6), which differed in the length of the amino acid spacer placed in position 2, were prepared coupling Fmoc-glycine, Fmoc-β-alanine, Fmoc-γ-aminobutyric acid, Fmoc-5-aminovaleric acid, Fmoc-ε-aminocaproic acid and Fmoc-7-aminoeptanoic acid to different aliquots of Hn peptidyl-resin. After coupling of the appropriate linker, a cysteine residue was added at the N-terminus. The resin cleavage and HPLC purification afforded the final fully deprotected Hn peptides (Supplementary Table S1).

Chemical ligation reactions between purified AcHx(thioester) and Hn(0–6) peptides were performed in ligation buffer (0.2 M sodium phosphate pH 7.3, 4 M guanidinium-HCl, 50 mM 4-mercaptophenylacetic acid, 20 mM Tris(2-carboxyethyl)phosphine hydrochloride, 2 mM ethylenediaminetetraacetic acid)^33^ mixing AcHx(thioester) and cysteinyl-Hn peptides in a 1:5 molar ratio. Ligation reaction was performed at room temperature under mild stirring for 16 h until completion as judged by HPLC trace at 210 nm. Then, ligation mixtures were dialyzed against water, lyophilized and purified by reverse-phase HPLC, affording the seven final peptides variants (from EP0 to EP6) with purity >95% as determined by HPLC analysis (Supplementary Fig. S3). EP peptides identity was confirmed by mass spectroscopy (Supplementary Table S1, Supplementary Fig. S3).

### VEGF receptor phosphorylation and signaling

**Figure 2:**
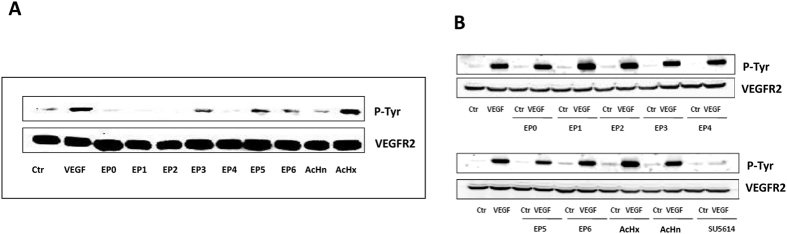
Effect of EP peptides on VEGFR2 phosphorylation. Western blot analysis performed using anti-phospho-Tyr antibody on cellular membranes of (**A**) PAEC overexpressing VEGFR2 stimulated with VEGF or with EP0-6 peptides (50 ng/mL each, 15 min at 37 °C) (western blot quantification expressed as fold increase respect to Ctr: VEGF 6.5 ± 1.3, EP0 0.7 ± 0.25, EP1 0.75 ± 0.09 EP2 0.5 ± 0.18, EP3 4.1 ± 1.3 EP4 0.7 ± 0.16, EP5 4.7 ± 1.2, EP6 4.8 ± 1.8, AcHn 0.9 ± 0.08, AcHx 4.7 ± 0.9) and (**B**) PAEC overexpressing VEGFR2 pretreated with 50 ng/mL of EP0-6 peptides for 15 min and then with 50 ng/mL of VEGF for additional 15 min in serum deprivation condition (Ctr). AcHn and AcHx peptides (50 ng/mL) and the tyrosine kinase inhibitor SU5614 (10 μM) were used as controls. Anti-VEGFR2 antibody was used as loading control. Gels are representative of three experiments (western blot quantification expressed as ∆ of fold increase respect to VEGF induced response: EP0 0.89 ± 0.08, EP1 1 ± 0.05, EP2 0.95 ± 0.04, EP3 0.76 ± 0.18, EP4 0.79 ± 0.23, EP5 1.12 ± 0.04, EP6 1.2 ± 1.3, SU5614 0.15 ± 0.05).

VEGF binds and activates its receptors by inducing VEGFR auto-phosphorylation on specific Tyr residues^20^. We evaluated the biological activity of EP peptides analyzing their ability to modulate VEGFR2 auto-phosphorylation. Porcine aortic endothelial cells (PAEC) overexpressing VEGFR2^34^ were cultured in starvation medium and treated with VEGF or with EP peptides (50 ng/mL each, 15 min). After treatment, cell membranes were analyzed by immunoblotting using an antibody raised against phospho-tyrosine^35^. AcHn and AcHx, mimicking each single epitope reproduced by EP peptides, were used as controls (50 ng/mL). VEGF, EP3, EP5, EP6 and the control peptides AcHn and AcHx induced VEGFR2 phosphorylation (Fig. 2A). When PAEC-VEGFR2 were exposed to EP peptides (50 ng/mL) for 15 minutes and then to VEGF (50 ng/mL for additional 15 min), neither EP nor control peptides were able to antagonize VEGF-stimulated phosphorylation of VEGFR2, which was instead inhibited by the tyrosine kinase inhibitor SU5614 (10 μM)^36^ (Fig. 2B). Based on these results, we decided to better characterize the biological activity profile of peptides EP3, EP5 and EP6.

**Figure 3:**
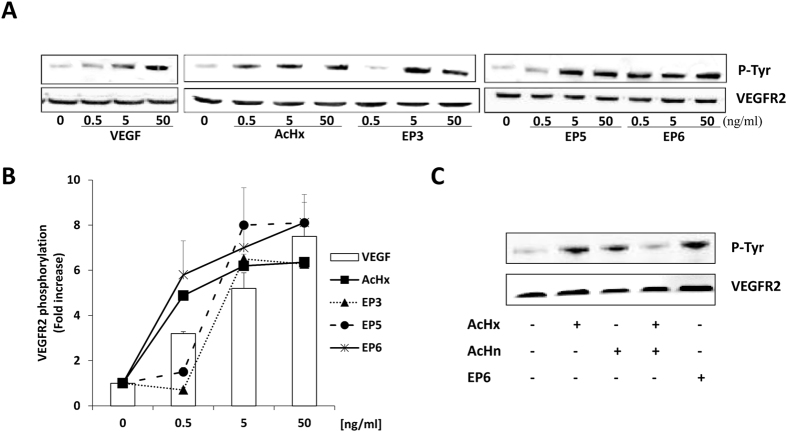
Comparison of EP and control peptides on VEGFR2 autophosphorylation. (**A**) Dose-response of VEGFR2 phosphorylation by the selected EP peptides (EP3, EP5 and EP6). PAEC overexpressing VEGFR2 were treated with increasing concentrations (0.5, 5 and 50 ng/mL) of VEGF, EP3, EP5 and EP6 peptides (15 min at 37 °C). AcHx peptide was used as control. (**B**) Western blot quantification. Data in the graph report VEGFR2 phosphorylation increase respect to cells in serum deprivation condition (n = 3). (**C**) Comparison of the effect of EP6 and control peptides, tested alone and in combination, on VEGFR2 phosphorylation. PAEC-VEGFR2 were exposed to EP6 (50 ng/mL), AcHx (22 ng/mL), AcHn (26 ng/mL) and their combination for 15 min. Cell lysates were analyzed by immunoblotting using anti-phosphotyrosine antibody. Anti-VEGFR2 antibody was used as loading control. (western blot quantification expressed as fold increase respect to Ctr: AcHx 4.9 ± 1.3, AcHn 5.1 ± 0.95, combination 1.4 ± 0.89, EP6 5.4 ± 1.1).

We verified if the selected peptides could stimulate VEGFR2 phosphorylation in a dose-dependent manner. Western-blot analysis of PAEC cells samples incubated with increasing concentrations (0.5, 5 and 50 ng/mL) of EP3, EP5 and EP6 showed that all peptides stimulated VEGFR2 phosphorylation in a dose-dependent manner (Fig. 3A,B). EP6 was the most potent molecule, being able to promote maximal fold increase of VEGFR2 phosphorylation at the lowest concentration assayed (0.5 ng/mL). The equimolar comparison of EP6 with AcHx and AcHn induced responses documented the higher efficacy of EP6 respect to the control peptides. Moreover, the simultaneous exposure to the two control peptides abrogated VEGFR2 autophosphorylation (Fig. 3C).

**Figure 4:**
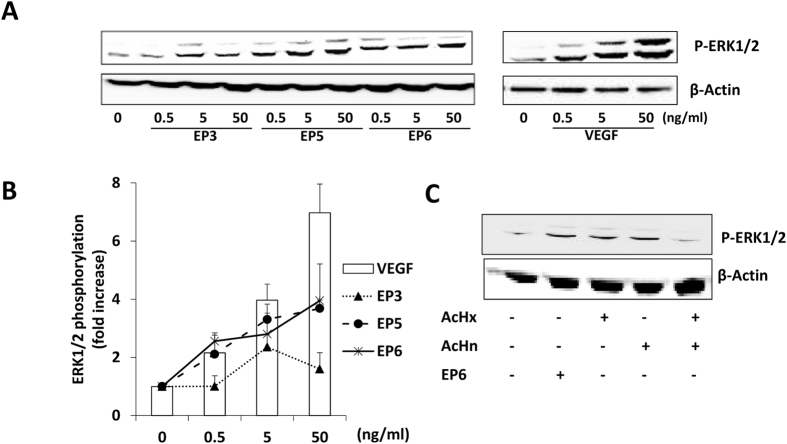
ERK1/2 pathway activation analysis. (**A**) PAEC-VEGFR2 were incubated for 15 min at 37 °C in starvation medium with different concentration (0.5, 5 and 50 ng/mL) of EP3, EP5, EP6 peptides and VEGF. (**B**) Data in the graph report ERK1/2 phosphorylation increase respect to cells in serum deprivation condition (n = 3). (**C**) Comparison of the effect of EP6 and control peptides, tested alone and in combination, on ERK1/2 phosphorylation. PAEC-VEGFR2 were exposed to EP6 (50 ng/mL), AcHx (22 ng/mL), AcHn (26 ng/mL) and their combination for 15 min. Cytosolic extracts from treated cells were analyzed by Western blot using anti-phospho ERK1/2. Anti-β-actin antibody was used as loading control (western blot quantification expressed as fold increase respect to Ctr: EP6 3.8 ± 0.4, AcHx 3.1 ± 0.9, AcHn 2.9 ± 0.9, combination 1.9 ± 0.75).

It is well established that VEGF binding to receptors on ECs induces the activation of MAPK-ERK1/2 pathway^37^, resulting in cell proliferation and inhibition of cell apoptosis. We therefore investigated whether EP peptides were able to induce ERK1/2 phosphorylation on ECs^35^. ERK1/2 phosphorylation was induced in a concentration dependent manner by EP5 and EP6 (Fig. 4A,B) peptides, confirming the VEGF-like behavior of the selected molecules. The equimolar comparison of EP6 and control peptides documented that EP6, AcHx and AcHn similarly activated ERK1/2 in ECs, while the combination of the two control peptides failed to reproduce the EP6 cellular response (Fig. 4C). It seems that the concomitant action of the two isolate peptides (AcHn and AcHn) prevents VEGFR activation, probably failing to induce receptor dimerization; instead if the binding regions (Hn and Hx) are constrained together with the right linker (EP6) a VEGF-like activity is showed.

### Effect of EP peptides on ECs proliferation

**Figure 5:**
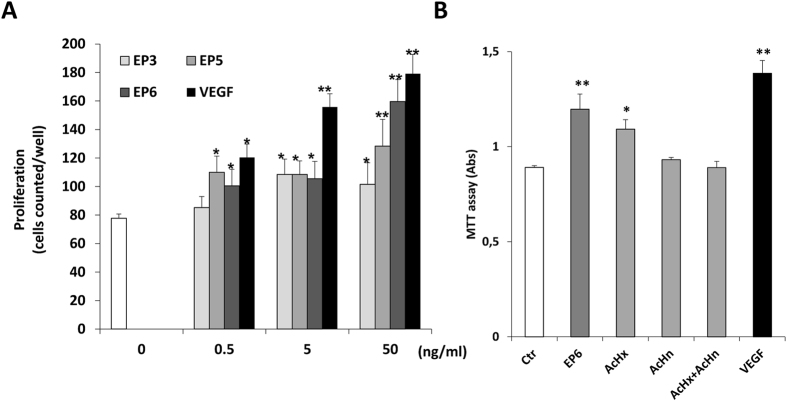
Effect of EP peptides on EC proliferation and survival. (**A**) Subconfluent HUVEC cultures were incubated in starvation medium with the indicated concentrations of EP3, EP5 and EP6 for 72 h and then counted. VEGF was used as a positive control. P < 0.05 and **P < 0.01 vs basal control. (**B**) PAEC-VEGFR2 were stimulated with EP6 (50 ng/mL), AcHx (22 ng/mL), AcHn (26 ng/mL) and their combination, or VEGF (50 ng/mL) for 48 h. Cell survival was measured by MTT assay and data are reported as absorbance units (n = 3).

As the activation of ERK1/2 pathway in ECs leads to DNA synthesis and cell proliferation^38^, we evaluated whether EP3, EP5 and EP6 peptides were able to stimulate cell growth. A proliferation assay was performed on human umbilical vein endothelial cells (HUVECs). Cells in starvation medium were incubated with increasing concentrations (0.5, 5 and 50 ng/mL) of the selected peptides and VEGF, and cell number was counted after 72 h. All EP peptides induced cell growth in a dose-dependent manner, being EP5 and EP6 the most effective molecules (Fig. 5A). Cell survival was evaluated by MTT assay in PAEC-VEGFR2 following stimulation with EP6 and equimolar concentration with control peptides for 48 h. The results, reported in Fig. 5B, document that EPαrosurvival activity is higher than AcHx and AcHn tested alone or in combination.

The same proliferation assay was repeated in HUVEC in presence of the tyrosine kinase inhibitor SU5614 (Table 1). In the presence of the inhibitor, the proliferative effect of the EP peptides was suppressed, confirming that EP-induced proliferation was correlated to VEGFR2 phosphorylation.

### Effect of EP peptides on ECs migration

**Figure 6:**
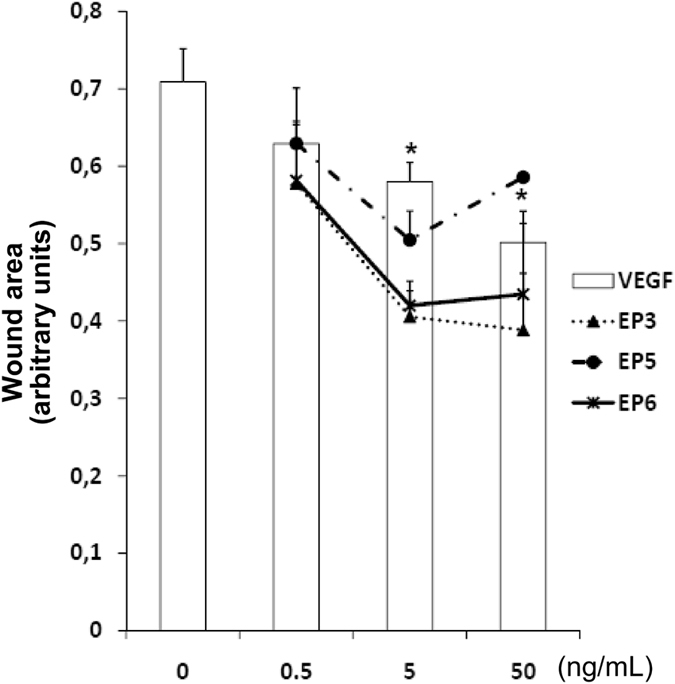
Effect of EP peptides on EC migration. HUVEC monolayers were scratched with a pipette tip to mimic a wound. Adherent cells were then treated with increasing concentrations of EP3, EP5 and EP6 (0.5, 5 and 50 ng/mL), in the presence of the cell proliferation inhibitor ARA-C (2.5 μg/mL). VEGF was used as control. Wound area was measured after 18 h of incubation by image analysis. *p < 0.05 vs basal control. Data are expressed as arbitrary units of wound area taking as reference the area at time 0.

The ability of EP peptides to induce EC migration was evaluated performing a scratch assay on confluent HUVECs (Fig. 6). After having produced a scratch on cell monolayers, cells were exposed to increasing concentrations (0.5, 5 and 50 ng/mL) of VEGF and EP3, EP5 and EP6 at 37 °C, in presence of the antimitotic agent ARA-C (2.5 μg/ml)^39^. Wound healing was evaluated measuring wound area in time-course by microscopy imaging. Data reported in Fig. 6 refers to wound area measured after 18 h of incubation with the assayed molecules. All the peptides were able to stimulate EC migration. EP3 and EP6 peptides outstand as the most effective molecules.

### *In vitro* angiogenesis assay

**Figure 7:**
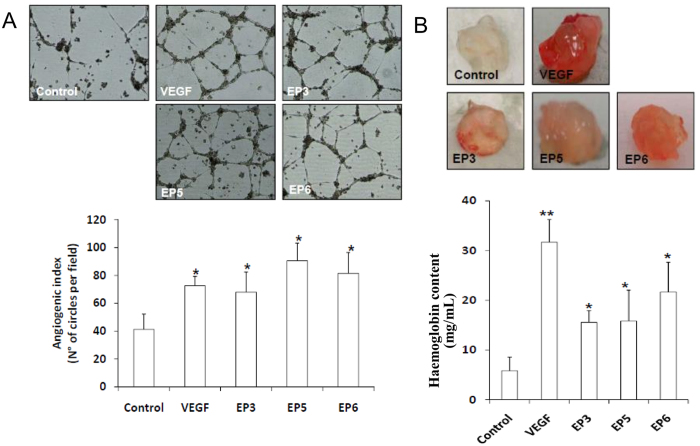
*In vitro* and *in vivo* angiogenic properties of EP peptides. (**A**) HUVECs were plated onto a layer of basement membrane matrix (Matrigel) and incubated at 37 °C for 18 h in the presence of EP3, EP5, EP6 and VEGF (50 ng/mL). After treatment, photomicrographs of tubular structures (Top) were quantified as angiogenic index, calculated as the number of complete circles counted/field by microscope image analysis (Bottom). *p < 0.05 vs basal control. (**B**) *In vivo* Matrigel angiogenesis assay was performed by injecting Matrigel solution containing 500 ng of the selected EP peptides into the dorsal midline region of C57 black mice. Matrigel plugs loaded with VEGF (500 ng) or buffer were respectively used as positive and negative controls. After 10 days Matrigel implants were recovered (Top) and analyzed for their haemoglobin content (bottom). *p < 0.05 and **p < 0.01 vs Matrigel alone.

To investigate whether EP peptides were able to reproduce the overall angiogenic properties of VEGF, we evaluated their ability to induce ECs network formation on a Matrigel substrate (Fig. 7A). HUVECs were seeded on a Matrigel layer and stimulated with 50 ng/mL of EP3, EP5, EP6 or VEGF for 18 h. HUVECs structurally reorganize in tubules and networks (Fig. 7A, *Top*), whose extent was estimated by calculating the the angiogenic index as the number of circles counted *per* field^40^ (Fig. 7A, *Bottom*). All tested peptides induced the formation of new connections between ECs in Matrigel to an extent similar to that observed for VEGF. In order to check if EP6 peptide showed a functional improvement with respect to the control peptide AcHx, the *in vitro* angiogenesis assay was performed using equimolar amount of the two peptides (Supplementary Fig. S4). AcHx did not induce the pseudocapillary formation, reinforcing the idea that constraining the two VEGF binding regions will provide a more functional molecule.

### *In vivo* angiogenesis assay

The pro-angiogenic activity of EP peptides was evaluated by an *in vivo* test, using Matrigel plugs loaded with EP3, EP5 or EP6 peptides or VEGF (500 ng/plug). Matrigel solutions containing the test molecules were injected in the flank of mice and recovered after 10 days. In VEGF- and EP-containing implants blood vessel formation was observed (Fig. 7B, *Top*). The hemoglobin content of explanted plugs was measured by a spectrophotometric assay (Fig. 7B, *Bottom*). This experiment confirmed that EP peptides, and in particular EP6, display a VEGF-like activity, promoting angiogenesis and neovascularization *in vivo*.

### Circular dichroism spectroscopy

The conformational preferences of the designed peptides were analyzed in solution by circular dichroism. Peptides were dissolved in 5 mM phosphate buffer pH 7 and analyzed in the far UV spectrum region. All peptides showed a CD spectrum with a minimum around 200 nm typical of a disordered structure (Supplementary Fig. S5). Successively, we performed a titration using trifluoroethanol, a co-solvent with secondary-structure stabilizing effect^41^,^42^, to verify if the peptides had a tendency to adopt a specific secondary structure. Increasing trifluoroethanol concentration from 0–40% changed the CD spectrum of all peptides except for AcHn peptide, which remained substantially unmodified (Supplementary Fig. S5b). The minimum at 200 nm progressively shifted towards 208 nm and a second minimum appeared around 220 nm. These spectra variations are interpreted as a progressively increase of the peptide helical content^43^. The CD spectra of EP6 peptide (Supplementary Fig. S5c) is showed as representative of the EP peptides.

### Interaction of EP6 with VEGFR1D2 by NMR spectroscopy

A NMR analysis was performed to gain insight on the molecular basis of the EP6 interaction with VEGF receptor. In particular, we analyzed the interaction between EP6 and the second domain of VEGFR1 (VEGFR1D2)^44^, which possesses the major binding determinant for VEGF^21^, in analogy to our previous work on the characterization of the VEGF mimetic peptides^22^,^25^,^45^,^46^.

^1^H NMR spectrum (Supplementary Fig. S6a) and a combination of 2D [^1^H, ^1^H] TOCSY and 2D [^1^H, ^1^H] NOESY spectra (Supplementary Fig. S7a) of EP6 were acquired in Tris–HCl buffer and used to obtain the sequence specific proton resonance assignment (Supplementary Table S2).

**Figure 8:**
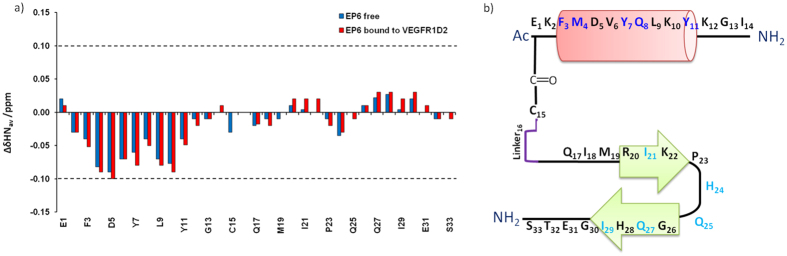
EP6 secondary structure. (**a**) Plot of the differences between the observed alpha proton chemical shifts and the corresponding random coil values (∆δHα) versus the amino acid sequence of free EP6 (blue) and of EP6 in the presence of VEGFR1D2 (red). (**b**) Sequence representation of EP6 secondary structure. Residues showing the biggest STD effects are highlighted in blue while those showing lower STD effects are highlighted in cyana.

The chemical shift assignment allowed an analysis of the secondary structure of EP6 by comparison with random coil values. As shown in Fig. 8a, the Hα proton chemical shift are all included within ±0.1 ppm from random coil values. Nevertheless, residues 3–11 present a uniform behavior indicating a clear α-helical secondary structure propensity. Accordingly, the assigned NOEs are mainly intra-residue and the few sequential connectivities do not indicate the presence of a stable secondary structure, in agreement with the CD analysis (Supplementary Fig. S5).

Successively, we analyzed the structural changes on EP6 induced by VEGFR1D2 performing transferred NOESY experiments. Addition of VEGFR1D2 to EP6 solution, in a 1:10 ratio, induced a soft broadening of almost all the proton resonances of the peptide, associated with small, but significant changes of the chemical shifts (Supplementary Table S3, Supplementary Fig. S6b). The comparison of the chemical shift perturbations indicates that the interaction with VEGFR1D2 induces an increase of EP6 secondary structure. Particularly, α-helix is further stabilized but, more interestingly, residues 20–30 tends to fold in a β-hairpin conformation (Fig. 8a). Accordingly, a significant improvement, in term of number and intensity of NOE cross-peaks, of the NOESY spectrum of EP6 in complex with VEGFR1D2 with respect to the NOESY spectra of free EP6 (Supplementary Fig. S7) are observed. In particular, in the presence of VEGFR1D2, several medium range αN (i, i + 3) and αβ (i, i + 3) trNOEs are detected along the peptide sequence for the region encompassing residues 3–11 (Supplementary Table S4), confirming the high helical propensity of this region of EP6. Furthermore, a few number of long range HN-HN (between residues Arg20 and His28, Lys22 and Gly26) and Hα-HN interactions (between residues Arg20-Ile29, Ile21-His28, Lys22-Gln27) are detected supporting the presence of a short two-stranded β-sheet encompassing the EP6 amino acid sequence 20–22 and 27–29 (Supplementary Table S4). Thus, the VEGFR1D2-induced conformation of EP6 is defined by a helix at the N-terminal (residues 3–11) followed by a β-hairpin at C-terminal (residues 20–29) (Fig. 8b).

**Figure 9:**
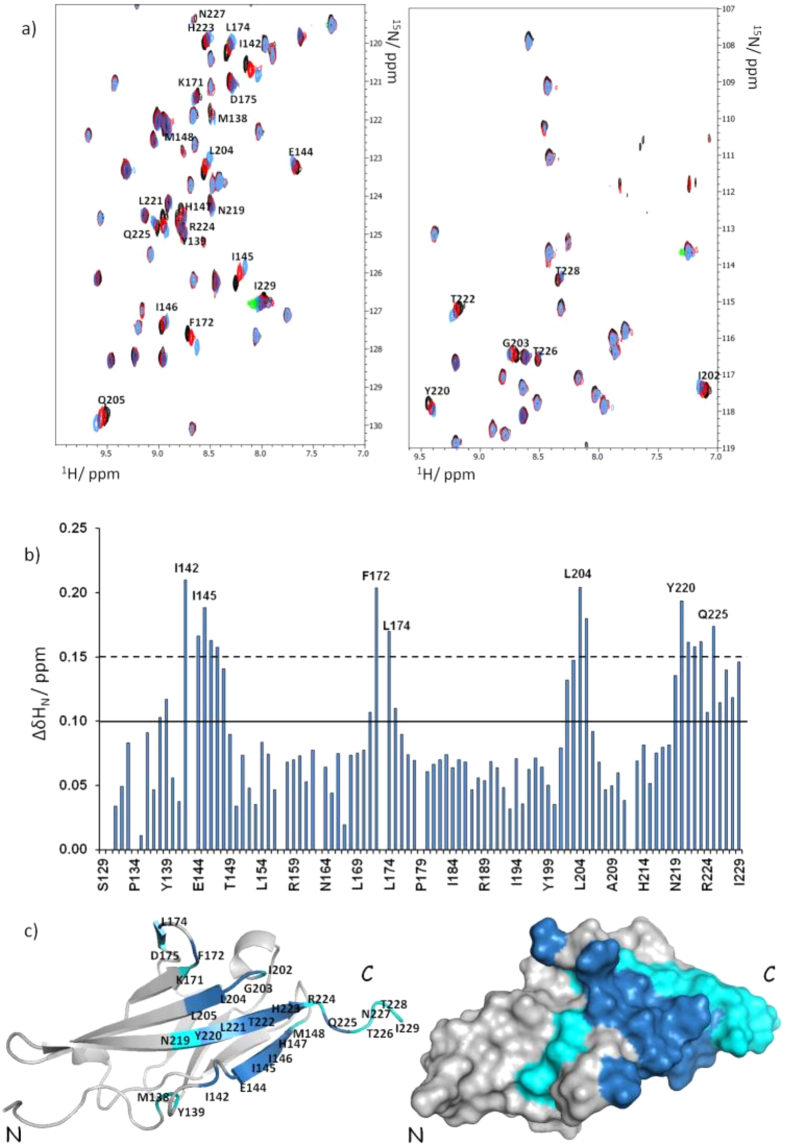
Chemical shift perturbation studies of VEGFR1D2 with EP6. (**a**) Superposition of two 2D [^1^H,^15^N] HSQC sections of VEGFR1D2 in the absence (black), in the presence of 1 equivalent (red) or 5 equivalents of EP6 (blue). The most relevant shifts observed in the amide cross-peaks are indicated. (**b**) Bar graphs of the average combined chemical shift differences (∆δHNav) as a function of sequence. The mean value is shown as a continuous line (∆δHNav ≥ 0.10 ppm); the mean value plus standard deviation is shown as a broken line (∆δHNav ≥ 0.15 ppm). (**c**) Chemical shift mapping onto one representative model of the VEGFR1D2 NMR structure (PDB ID: 1QSV) shown as ribbon (left) and as solvent-accessible surface (right). Residues for which 0.010 < ∆δHNav < 0.015 are shown in cyana; residues for which ∆δHNav > 0.15 are shown in sky blue. Figure was prepared using the software PYMOL (The PyMOL Molecular Graphics System, Version 1.8 Schrödinger, LLC).

To identify VEGFR1D2 residues involved in the interaction with EP6, an NMR chemical shift perturbation (CSP) analysis was performed by acquiring 2D [^15^N, ^1^H] HSQC spectra using ^15^N labeled VEGFR1D2 in the absence and presence of unlabeled EP6 peptide. Upon progressive addition of EP6 continuous changes in ^1^H and ^15^N chemical shifts for several VEGFR1D2 signals were observed (Fig. 9a) indicating the formation of VEGFR1D2/EP6 complex in a fast exchange regime between free and bound protein. To quantify CSPs, averaged combined chemical shift difference (ΔδHN_av_) of the amide cross-peaks in the presence and absence of EP6 were determined and plotted versus the residue number (Fig. 9b). On these bases, receptor residues involved in the peptide recognition have been identified: those having the largest ΔδHNav (>0.15 ppm, given by the mean value plus SD) are mainly located in the N-terminal βa strand region (I142), in βa’ strand (E144, I145, H147), in βf strand (L204, L205), in βg strand (Y220, L221, H223, Q225, I229), in the loop separating βc and βc’ (F172, L174). In addition, residues having 0.010 < ΔδHNav < 0.015 constitute two protein regions contiguous to that outlined by the most perturbed residues.

Mapping of these two sets of residues onto the NMR structure of protein VEGFR1D2 (1QSV.pdb) (Fig. 9c) reveals that the residues most perturbed by the addiction of EP6 are located on a contiguous and rather extensive interaction surface. In particular, this surface is defined by β*a’*, β*f*, and β*g* strands, the β*c*-β*c’* loops and the N-terminal portion, suggesting that it represents the binding site for EP6. In addition, we estimated the dissociation constant (K_D_) for the VEGFR1D2/EP6 complex. The overall apparent K_D_ for the residues (Supplementary Fig. S8) included in the VEGFR1D2 binding site is an average value and resulted to be 139 ± 38 μM. This analysis confirms that the *in vitro* peptide-receptor interaction occurs in a NMR fast-exchange regime. The molecular conditions existing on the cell surface, such as the receptor dimerization and crowding, are certainly very different, and very likely reflecting in a stronger *in vivo* binding constant as biochemical data clearly suggest. The VEGFR1D2 surface utilized for EP6 recognition, here identified by CSP studies, mainly corresponds to the binding surface used by VEGF for interaction with the receptor.

Successively, we performed Saturation Transfer Difference (STD) experiments, in order to gain insight on the EP6 residues involved in the binding to VEGFR1D2. In particular, 2D STD TOCSY spectra were carried out to facilitate unambiguous identification of VEGFR1D2-binding residues^47^. The Hβ proton resonances of Phe3, Tyr7 and Tyr11 delineate intense STD effects, indicating their close contact with the protein (Supplementary Fig. S9). Similarly, strong STD effects were also detected for Gln8 Hβ and Hγ of and for M4 Hβ. On the other hand, lower degree of saturations were observed for the side chain resonances of residues localized onto the β-hairpin region, as well as for the aliphatic methyl protons of Ile21 and Ile29, the Hβ protons of the His 24, the Hβ and Hγ protons of Gln25 and Gln27, indicating their proximity to VEGFR1D2. Finally, NMR binding studies suggest that intermolecular interactions between EP6 and VEGFR1D2 include a network of hydrophobic interaction contacts. Most of these stabilizing intermolecular interactions involve the aromatic residues Trp3, Tyr7 and Tyr11 and hydrophobic residues such as Ile 21 and Ile 29 of the peptide and I142, I145, F172, L174, L204, Y220 and L221 residues on the VEGFR1D2 surface.

## Conclusions

Peptides reproducing a portion of the discontinuous VEGFR-binding site of VEGF were designed and synthesized by chemical ligation. The peptides covalently join two VEGF regions involved in the receptor recognition located on different monomers. A functional and biological characterization *in vitro* on endothelial cells and *in vivo* disclosed the most active peptide and revealed a VEGF-like behavior. A detailed NMR analysis in presence of the second domain of VEGF receptor highlighted the molecular determinants of the peptide-receptor interaction and showed that the receptor binding induces a conformational variation in the peptide conformation, providing essential information for the development of more active VEGF-receptor binding molecules.

## Methods

Peptide synthesis and protein expression and purification are reported as Supplementary Methods.

### Cell line and culture conditions

Human Umbilical Vein Endothelial Cells (HUVECs) were purchased from Lonza (Basel, Switzerland). All experiments were performed on low passage cell cultures. Cells were grown in Endothelial Growth medium (EGM-2) (EBM-2, FBS 10%, VEGF, R^3^-IGF-1, hEGF, hFGF, hydrocortisone, ascorbic acid, heparin and GA-1000) (Clonetics, Cambrex Bio Science Walkersville, Walkersville, USA) at 37 °C and in 5% CO_2_.

Porcine aortic endothelial cells (PAEC) overexpressing vascular endothelial growth factor receptor-2 (KDR)^34^ were maintained with Ham’s F-12 medium supplemented with 10% FBS and 500 μg/ml G418 sulfate antibiotic. PAEC-KDR cells were split weekly 1:3.

### Cell proliferation assay

HUVECs were plated at density of 1500 cells/well in 96-well gelatin-coated microplates. After 4 h incubation, cells were treated with VEGF_165_ (R&D Systems, Minneapolis, MN, USA) or EP peptide (from 0.5 to 50 ng/mL) for 72 h. Where reported, SU5614 (10 μM) (Calbiochem, Milan, Italy) was administered 30 min before the treatment with the tested peptides. Cell proliferation was evaluated as number of cells counted per well^35^.

### Cell survival

PAEC-VEGFR2 were seeded in Ham-F12 (10% serum) and plate at the density of 1500/well of a 96 multiwell plate. After adhesion, cells were treated with EP6 (50 ng/mL), AcHx (22 ng/mL), AcHn (26 ng/ml), their combination and VEGF (50 ng/mL). After 44 h, medium was removed and cells were incubated for 4 h with fresh medium in the presence of 1.2 mM MTT (3-(4,5-dimethylthiazol-2-yl)-2,5-diphenyltetrazolium bromide). Living cells reduce MTT to a strongly pigmented formazan product. After solubilization in DMSO, absorbance of the formazan was measured with a microplate absorbance reader (Tecan, San Jose, CA, USA) at 540 nm. Data are expressed as absorbance units (Abs).

### Wound healing model assay

HUVECs were plated in 24-well plates at 1 × 10^5^ cells/well and incubated for 24 h to became confluent. Cell monolayers were scored vertically down the center of each well with a sterile tip. Each well was washed with PBS to remove detached cells. Fresh media containing the test substances was complemented with ARA C (Sigma-Aldrich, Milan, Italy) (2.5 μg/ml) to inhibit cell proliferation. Images of the wound in each well were acquired at time 0 and after 18 h under phase contrast microscope at the magnification of 10X. Data are reported as % of wound area^25^.

### Immunoblotting for phospho-VEGFR2 and phospho-ERK1/2

Fast activation of VEGFR2 and ERK1/2 was evaluated by western blotting as previously described^35^. Sparse and serum starved PAEC- VEGFR2 were treated for 15 min with different concentration of VEGF or EP peptides (from 0.5 to 50 ng/mL). Where reported, the inhibitor SU5614 (10 μM) was added 15 minutes before the treatment with the peptides. Samples were obtained by the use of lysis buffer with the following composition: 50 mM Tris-HCl, 1% Triton-X, 1 mM Na_3_VO_4_, 1 mM EGTA, 0.2 mM PMSF, 25 μg/ml leupeptin, 10 μg/ml aprotinin, 10 mM NaF and 150 mM NaCl. After centrifugation the supernatant was recovered and proteins were assayed. Electrophoresis was carried out in SDS 10% polyacrylamide gel. Proteins were then blotted onto activated nitrocellulose membranes, incubated overnight with the antibodies anti-phospho-Tyr or anti phospho-ERK1/2 (Cell Signalling Technology, Euroclone, Pero (MI), Italy) diluted 1:1000 in PBS containing 1% dried milk and 0.05% Tween 20 and then detected by enhanced chemiluminescence system (Bio-Rad, Milan, Italy). Results were normalized to those obtained by using an antibody raised against KDR (Millipore, Milan, Italy) or antibody anti-beta-Actin (Sigma-Aldrich, Milan, Italy).

### *In vitro* angiogenesis model

HUVEC were plated onto a thin layer (300 μL) of basement membrane matrix (Matrigel; Becton Dickinson, Waltham, MA, USA) in 24-well plates at 6 × 10^4^ cells/well in EBM (containing 2.5% FBS) and incubated at 37 °C in 5% CO_2_ for up 18 h in the presence of test substances. Quantification of tubular structures and complete circles was performed on digitalized photomicrographs as previously described^40^.

### *In vivo* Matrigel angiogenesis assay

*In vivo* Matrigel angiogenesis assay was performed as previously described^39^. C57 black mice (20–25 g) were supplied by Charles-River (Calco (LC), Italy) and kept in temperature- and humidity-controlled rooms (22 °C, 50%) with lights on from 07:00 to 19:00 h, water and food available ad libitum. All procedures were carried out in accordance with the Italian law (Legislative Decree no.116, 27 January 1992), which acknowledges the European Directive 86/609/EEC. All efforts were made to minimize the number of animals used and their suffering. VEGF or EP peptides were diluted in Matrigel (Becton Dickinson, growth factors and phenol red-free) on ice to a final concentration of 500 ng. Mice were subcutaneously injected in the dorsal midline region with 0.3 ml of Matrigel alone or with Matrigel containing the stimuli. After 10 days mice were euthanized and implants recovered. Plugs were resuspended in 1 ml of Drabkin’s reagent for 18 h on ice and haemoglobin concentration was determined by absorbance at 540 nm and compared with a standard curve (Sigma-Aldrich, Milan, Italy).

### Statistical analysis

Statistical analysis was performed using GraphPad Prism version 4.00 for Windows (GraphPad Software, La Jolla, CA, USA). Results are reported as the mean with SD of at least three experiments run in three to five replicates. Comparisons between control and treated samples were made with a paired Student’s t test.

### Circular dichroism spectroscopy

CD spectra were collected on a Jasco 720 (Easton, MD, US) instrument in the range 185–260 nm using a 1 mm path-length quartz cuvette (Hellma, Milan, Italy) at 20 °C and setting the following parameters: scanning speed 10 nm/min, bandwidth 2.0 nm, data pitch 0.2 nm, response 4 s, and three spectra accumulations. Peptides were analyzed in 5 mM phosphate buffer pH 7 with increasing concentration of trifluoroethanol (Romil, Cambridge, UK) and peptide concentrations ranging from 50 μM to 100 μM. Peptides were dissolved in filtered and degassed water and then diluted to the final concentration using the appropriate volume of 10x buffer solution, water and TFE. Peptide concentrations were determined by absorbance at 280 nm^48^ using a molar extinction coefficient of 2980 M^−1^ cm^−1^. CD spectra are displayed in molar ellipticity.

### NMR samples

1.3 mg of EP6 peptide was dissolved in 600 μL of 20 mM Tris–HCl (pH 7.5), 0.02% sodium azide and 10% ^2^H_2_O. For titration of VEGFR1D2 with EP6, ^15^N-labeled VEGFR1D2 was dissolved in 600 μL at 110 μM concentration in 20 mM Tris–HCl (pH 7.5), 0.02% sodium azide and 10% ^2^H_2_O. EP6 was added to the VEGFR1D2 solution as increments of known amounts of lyophilized peptide dissolved at higher concentration to obtaining peptide final concentrations ranging from 27.5 to 550 μM and a protein final concentration of 110 μM. For STD experiments^49^, 55 μM final concentration of VEGFR1D2 was added at 550 μM of EP6 in 20 mM Tris–HCl (pH 7.5), 0.02% sodium azide and 10% ^2^H_2_O (1:10).

### NMR spectroscopy

All NMR experiments were recorded at 298 K on a Inova 600 MHz spectrometer (Varian Inc., Palo Alto, CA, USA), equipped with a cryogenic probe optimized for ^1^H detection. All two-dimensional (2D) spectra were acquired in phase-sensitive mode using the time-proportional phase-incrementation (TPPI) method^50^ to obtain quadrature detection in the t_1_ dimension.

For chemical shift perturbation (CSP) studies, 2D [^15^N, ^1^H] heteronuclear single quantum coherence (HSQC) spectra were acquired with 1024 (HN) × 128 (N) data points and 8 scans. Spectral widths of 6714.8 and 2066.3 Hz were used in the HN and N dimensions, respectively. The data were apodized with a square cosine window function and zero filled to a matrix of size 4096 × 1024 before Fourier transformation and baseline correction. Water suppression was achieved using 3-9-19 pulse sequence with gradients^51^,^52^.

One-dimensional (1D) ^1^H spectra were acquired with a spectral width of 6712.0 Hz, relaxation delay 1.0 s, 7 k data points for acquisition and 16 k for transformation. For ^1^H chemical shift assignment of EP6, 2D [^1^H, ^1^H] total correlation spectroscopy (TOCSY)^53^ and nuclear Overhauser effect spectroscopy (NOESY)^54^ were acquired with 32 or 64 scans per t_1_ increment with a spectral width of 6712.0 Hz along both t_1_ and t2, 2048 × 256 data points in t_2_ and t_1_, respectively, and recycle delay 1.0 s. Water suppression was achieved by means of Double Pulsed Field Gradient Spin Echo (DPFGSE) sequence^55^,^56^. The TOCSY experiment was recorded using a DIPSI-2 mixing scheme of 70 ms with 7.7 kHz spin-lock field strength. The NOESY spectrum was carried out with a mixing time of 200 ms. The data were typically apodized with a square cosine window function and zero filled to a matrix of size 4096 × 1024 before Fourier transformation and baseline correction.

For protein–peptide interaction studies, 2D STD-TOCSY spectra were recorded with 128 increments in t_1_ and 32 transients using a MLEV-17 spin lock field of 70 ms. Saturation transfer was achieved by using 40 selective Gaussian pulses with a duration of 50 ms. STD spectra were recorded applying a 40 ms spin lock filter to eliminate the background protein resonances. Samples containing only the peptide did not show any STD effect, since the resulting difference spectrum did not contain any signal of the peptide. The 2D trNOESY experiments^57^ were performed at the molar ratio [VEGFR1D2]:[EP6] = 1:10 with three different mixing times (100, 150, 200 ms) at 298 K. trNOESY experiments were carried out with 456 increments in t1 and 2K data points in t_2_ using Double Pulsed Field Gradient Spin Echo (DPFGSE) sequence^55^,^56^ for the water suppression. After zero filling along t1 dimension, 4K(t2) X 1K (t1) data matrices were obtained.

All NMR data were processed with the software VNMRJ 1.1.D (Varian Inc.). 1D spectra were analyzed using ACD/NMR Processor 12.0 *(ACD/NMR Processor Freeware, Version 12.01 Advanced Chemistry Development, Inc., Toronto, ON, Canada (2012),* www.acdlabs.com). 2D [^1^H, ^1^H] TOCSY and NOESY spectra for ^1^H chemical shift assignment were analyzed using Neasy, a tool available in CARA (Computer Aided Resonance Assignment) software^58^ (downloaded from cara.nmr.ch). For CSP studies, 2D [^15^N, ^1^H] HSQC spectra were analyzed using Monoscope, a tool of CARA. Starting from the amide resonances for free VEGFR1_D2_, average combined chemical shift changes for bound VEGFR1D2 were determined using the following equation: ΔδHNav = [((ΔδH)^2^ + (ΔδN/5)^2^)/2]^½^, where ΔδH and ΔδN are the chemical shift variations of the amide proton and nitrogen resonances, respectively.

For determination of the dissociation constant of the VEGFR1D2/EP6 interaction, the absolute chemical shift changes for amide ^1^H (ΔδH = |δH_free_ − δH_bound_|) or ^15^N (ΔδN = |δN_free_ − δN_bound_|) of VEGFR1D2 in the free and bound forms were calculated. ΔδH or ΔδN values for residues whose Δδ was higher than the mean value were plotted as a function of total ligand concentration and fitted to a non-linear regression according to the equation for a two-state equilibrium model and a single binding site: Δδ = Δδmax ((K_D_ + [L] + [P]) − [(K_D_ + [L] + [P])^2^ − 4[P][L]]^½^)/2[P], where Δδmax is the total change in chemical shift at saturation, K_D_ is the dissociation constant, and [L] and [P] are the total ligand and protein concentrations at each titration point, respectively. Δδmax and K_D_ were used as fitting parameters. Fitting was performed using GraphPad Prism, v.5.00, (San Diego, CA, USA). Normalized shifts, Δδ/Δδmax, corresponding to the bound protein mole fraction, were also calculated and plotted against ligand concentration. The overall K_D_ was obtained as mean value (±standard deviation) over residues included in the EP6 binding site.

## Additional Information

**How to cite this article**: De Rosa, L. *et al.* Miniaturizing VEGF: Peptides mimicking the discontinuous VEGF receptor-binding site modulate the angiogenic response. *Sci. Rep.* **6**, 31295; doi: 10.1038/srep31295 (2016).

## Supplementary Material

Supplementary Information

## Figures and Tables

**Table 1:** Involvement of KDR in EP induced HUVEC proliferation.

	EP3		EP5		EP6	
	Basal	SU5614	Basal	SU5614	Basal	SU5614
**0**	77.8 ± 2.9	63.5 ± 8.7	77.8 ± 2.9	63.5 ± 8.7	77.8 ± 2.9	63.5 ± 8.7
**0.5 ng/mL**	85.2 ± 7.7	76 ± 11	110 ± 11	64 ± 8*	100 ± 11	70 ± 8*
**5 ng/mL**	108 ± 10	66.8 ± 7.1*	108.5 ± 9.4	76.3 ± 8.9*	106 ± 12	63.5 ± 9.5*
**50 ng/mL**	102 ± 11	75 ± 9*	128 ± 9	83 ± 12*	159 ± 15	57.4 ± 12.8*

The proliferative effect (cells counted/well) of the EP peptides was inhibited by preincubation with SU5614 (10 μM), denoting an involvement of VEGFR2 tyrosine kinase activation. *P < 0.01 vs agonist alone.
