# Supplementary material for: Miniaturizing VEGF: Peptides mimicking the discontinuous VEGF receptor-binding site modulate the angiogenic response
Source: Sci Rep. 2016 Aug 8;6:31295. doi: 10.1038/srep31295 (PMC4976335; doi:10.1038/srep31295)
Supplement: Supplementary Information [file srep31295-s1.pdf]

Supplementary Information

Miniaturizing VEGF: Peptides mimicking the discontinuous VEGF receptor-binding site modulate the angiogenic response

Lucia De Rosa, Federica Finetti, Donatella Diana, Rossella Di Stasi, Sara Auriemma, Alessandra Romanelli, Roberto Fattorusso, Marina Ziche, Lucia Morbidelli, Luca Domenico D'Andrea

Methods: Peptide Synthesis, VEGFR1D2 expression and purification,

Table S1: Calculated and experimental molecular weight of synthesized peptides

Table S2: ^{1}H chemical shift assignment of the free EP6 peptide

Table S3: ^{1}H chemical shift assignment of the bound EP6 peptide

Table S4: Nonsequential NOEs involving backbone protons of EP6 peptide in the presence of VEGFR1D2.

Figure S1: VEGF-VEGFR structural details.

Figure S2. Schematic representation of the synthetic protocol for EPs peptides

Figure S3: LC-MS analyses of purified EP0 – EP6 peptides.

Figure S4. In vitro angiogenic properties of EP6 and AcHx peptides

Figure S5: CD spectra of selected peptides.

Figure S6: ^{1}H NMR spectra of peptide EP6 free and in the presence of VEGFR1D2

Figure S7: A section of the 2D [^{1}H, ^{1}H] NOESY of free EP6 and 2D [^{1}H, ^{1}H] trNOESY spectrum of EP6

Figure S8: NMR binding studies of VEGFR1D2 with EP6.

Figure S9: 2D TOCSY spectrum of EP6 in presence of VEGFR1D2

Methods

AcHn, Hn0, Hn1, Hn2, Hn3, Hn4, Hn5, Hn6 peptide synthesis.

Hn peptides (Fig. 3) were synthesized on solid phase by standard Fmoc chemistry[1]. Hn peptides synthesis was carried out on the Rink Amide MBHA Resin LL with 0.38 mmol/g substitution grade (Novabiochem-Merck, Nottingham, UK) using Fmoc amino acids with standard protecting groups (Iris Biotech GmbH, Marktredwitz, Germany). Each reaction step was performed at room temperature under stirring. Deprotection steps were performed with 20% piperidine (Biosolve, Valkenswaard, The Netherlands) in dimethylformamide (DMF) (Romil, Cambridge, UK) (two incubation steps, 7 min each one). Each amino acid coupling reaction was performed twice using a five-fold molar excess of amino acid, 4.99 eq of 1-[Bis(dimethylamino)methylene]-1H-1,2,3-triazolo[4,5-b]pyridinium 3-oxid hexafluorophosphate (HATU) (GL Biochem, Shangai, China) and 10 equiv of *N*,*N*-diisopropylethylamine (Sigma-Aldrich, Milan, Italy). Each coupling step was carried out for 15 min. A capping step (5 min) was performed with a solution of 2 M acetic anhydride (Sigma-Aldrich, Milan, Italy), 0.06 M hydroxybenzotriazole (HOBt) (GL Biochem, Shangai, China), and 0.55 M DIPEA in 1-Methyl-2-pyrrolidinone (NMP) (Romil, Cambridge, UK) after each coupling step. After each reaction step, resin was washed in DMF (five washes of 1 min). Peptide cleavage from resin and deprotection were carried out using a mixture of trifluoroacetic acid (TFA) /triisopropylsilane (TIS) /H_2O /ethanedithiol (EDT) (Sigma-Aldrich, Milan, Italy) in the ratio 94/1/2.5/2.5 (v/v/v/v) for 3.5 h under stirring at room temperature. The resin was finally filtered and the crude peptide was precipitated in ice-cold diethyl ether (Sigma-Aldrich, Milan, Italy). Each peptide was purified by RP-HPLC performed on a AXIA RP-MAX Synergi column (4 μ, 80 Å, 50 x 21.2 mm; Phenomenex, Torrance, US) applying a linear gradient of CH_3CN (0.1% TFA) in H_2O (0.1% TFA) from 5% to 50% in 20 min using a flow rate of 20 mL/min. Pure peptides were finally lyophilized. Peptides purity and identity were verified by LC-MS on an Agilent 1200 Infinity Series (Agilent Technologies, Santa Clara, CA, US) chromatographic system equipped with a diode array combined with an electrospray ion source and a time-of-flight mass analyzer using a

C18 Jupiter column 150 x 2 mm, 300 Å, 3 µm (Phenomenex, Torrance, US) and applying a gradient of CH_3CN (0.1% TFA) in H_2O (0.1% TFA) from 5 to 70% in 20 min at a flow rate of 0.2 ml/min (Table S1).

AcHx and AcHx(thioester) peptide synthesis.

Hx peptide (Fig. 3) was synthesized by Fmoc chemistry using the resin Rink Amide PEG-MBHA 200-400 mesh (0.54 mmol/g, Iris Biotech GmbH, Marktredwitz, Germany). Fmoc deprotection was performed by washing the resin two times for 5 min with a solution of 30% piperidine in DMF. Coupling reactions were performed with 5 equiv of Fmoc-amino acid, 5 equiv of HATU and 10 equiv of DIPEA for 40 min at room temperature; capping steps (1x5 min) were performed with a solution of acetic anhydride (2 M), HOBt (0.06 M), and DIPEA (0.55 M) in NMP. Five washing steps with DMF (1 min each one) followed each reaction step. A Fmoc-Glu(OAll)-OH (Novabiochem-Merck, Nottingham, UK) residue was coupled at the N-terminus of Hx peptide.

Allyl deprotection. After deblocking and acetylation of the Nα-amino group, allyl-protecting moiety was removed from glutamic acid side chain in position 1 of Hx peptide. A solution of tetrakis(triphenylphosphine)palladium(0) (0.25 equiv) (Sigma-Aldrich, Milan, Italy) and phenylsilane (Sigma-Aldrich, Milan, Italy) (24 equiv) in dry dichloromethane (DCM, Sigma-Aldrich, Milan, Italy) was added to the resin under argon and shaken for 30 min. The procedure was repeated three times. The resin was subsequently washed with DCM.

Thioesterification reaction. Carboxylic group on the side chain of glutamic acid, exposed by allyl deprotection, was then thioesterified. 10 equiv of benzyl mercaptane (BzSH, Sigma-Aldrich, Milan, Italy) and then 10 equiv of N',N'-diisopropylcarbodiimide (DIC, Sigma-Aldrich, Milan, Italy) were added to the resin suspended in DCM. Reaction was left overnight, at room temperature and it was repeated once again. Thioester peptide was cleaved from resin and all the protecting groups removed by incubation in a solution of TFA/BzSH/H_2O/TIS (94/2.5/2.5/1 v/v/v/v) for 3 h. AcHx(thioester) peptide was precipitated in ice-cold diethyl ether and was finally purified by RP-HPLC on RP-MAX Synergi column (4 µ, 80 Å, 50 x 21.2 mm) using a linear gradient of CH_3CN

(0.1% TFA) in H_2O (0.1% TFA) from 10% to 60% in 20 min performed at 20 ml/min. Peptide purity and identity were verified by analytical RP-HPLC on an Agilent 1200 Infinity Series (Agilent Technologies, Santa Clara, CA, US) chromatographic system equipped with a diode combined with an electrospray ion source and a time-of-flight mass analyzer using a C18 Jupiter column 150 x 2 mm, 300 Å, 3 μm (Phenomenex, Torrance, US) and applying a gradient of CH_3CN (0.1% TFA) in H_2O (0.1% TFA) from 5 to 70% in 20 min performed at 0.2 ml/min (Tab. S1).

EP0, EP1, EP2, EP3, EP4, EP5 and EP6 synthesis by chemical ligation reactions.

Native chemical ligation reaction was carried out in 0.2 M sodium phosphate (Sigma-Aldrich, Milan, Italy) pH 7.3, 4 M guanidinium-HCl (Sigma-Aldrich, Milan, Italy), 50 mM 4-mercaptophenylacetic acid (Wako Chemicals, Neuss, Germany), 20 mM Tris(2-carboxyethyl)phosphine hydrochloride (Stremm Chemicals, Kehl, Germany), 2 mM ethylenediaminetetraacetic acid (Sigma-Aldrich, Milan, Italy). AcHx(thioester) and cysteinil-Hn peptides were dissolved in ligation buffer respectively at the final concentration of 0.1 mM and 0.5 mM (1:5 molar ratio). Ligation reaction was performed at room temperature under mild stirring for 16 h. Then ligation mixtures were dialyzed against water using a Spectra/Pore® membrane with a 1 KDa cut off (Spectrum, Rancho Dominguez, CA, US), lyophilized and purified by RP-HPLC on Jupiter C4 column 5 μ, 300 Å, 250 x 10 mm (Phenomenex, Torrance, US) using a linear gradient of CH_3CN (0.1% TFA) in H_2O (0.1% TFA) from 5% to 70% in 40 min performed at 5 mL/min. Peptide purity and identity was verified by analytical RP-HPLC on an Agilent 1200 Infinity Series (Agilent Technologies, Santa Clara, CA, US) chromatographic system equipped with a diode combined with an electrospray ion source and a time-of-flight mass analyzer using a C18 Jupiter column 150 x 2 mm, 300 Å, 3 μm (Phenomenex, Torrance, US) and applying a gradient of CH_3CN (0.1% TFA) in H_2O (0.1% TFA) from 5 to 70% in 20 min performed at 0.2 ml/min (Tab. S1 and Fig. S1). EPs peptides purity was > 95% based on the analytical HPLC area revealed at 210 nm.

VEGFR1D2 expression and purification

Reagents used for preparation of buffers and growth media of *Escherichia coli* and the reagents for polyacrylamide gels electrophoresis (Acrylamide, APS, TEMED, SDS, Tris, Glycine) were supplied by Sigma Aldrich (Milan, Italy), Euroclone (Pero, Italy), Applichem (Darmstadt, Germany) and MP Biomedicals (Santa Ana, CA, US). The molecular weight markers for proteins were from Sigma Aldrich and Applichem. The restriction and modification enzymes (calf intestine phosphatase and T4 DNA ligase) and *Taq* DNA polymerase (5 U/μL) were supplied by New England Biolabs. The molecular weight markers for nucleic acids were supplied by New England Biolabs (Ipswich, US) and Roche, while the *Pfu Turbo* polymerase (2.5 U/μL) was supplied by Agilent Technologies (Santa Clara, CA, US). The synthesis of the oligonucleotides was committed at Sigma-Genosys, while the sequencing service was committed at PRIMM srl; pETM11 *E. coli* expression plasmid was supplied by Novagen. DNA purification kits and Ni^{2+}-NTA resin were supplied by Qiagen. Purity and identity of protein samples were assessed both on a LCQ DECA XP MAX (Thermo Electron) liquid chromatography mass spectrometer equipped with an ESI source and an ion trap detector. The LC-MS analysis of proteins were carried out by RP-HPLC on a Jupiter C4 column (Phenomenex: 250 x 2.00 mm, 5 µm, 300 Å), using a linear gradient from 5 to 70% of CH_3CN (0.1%TFA) in water (0.1% TFA) in 30 min at a flow rate of 0.2 ml min^{-1}.

Expression, refolding and purification of human VEGFR1D2

VEGFR1D2 gene (DNA fragment encoding amino acids 129-229 of the human VEGFR1) was cloned as reported elsewhere[2]. For bacterial expression, the plasmid construct was transformed into E. *coli* BL21 *Codon Plus* (DE3) RIL cell strain (Agilent Technologies) carrying an inducible T7 RNA polymerase gene. Bacterial culture of 1L of prewarmed LB medium containing 50 µg mL^{-1} kanamycin and 33 µg mL^{-1} chloramphenicol was grown in shaking flasks at 37 °C until reaching 0.7/0.8 OD_{600}. Then, they were induced with 0.7 mM IPTG. After 4–5 h, the cells were harvested by centrifugation; the pellet was dissolved in 50 mM Tris-HCl, pH=8 containing protease inhibitors cocktail (Roche), to avoid proteins degradation, and the suspension was sonicated for 6 min, by using a Misonix Sonicator 3000 apparatus (Misomix, Farmingdale, US) with a micro tip probe and

an impulse output of 1.5/2 (=9/12 Watt). Bacterial lysate was then centrifuged (17000 rpm, 30 min, 4°C) and the supernatant (soluble fraction) and the pellet samples were analyzed by SDS-PAGE. The inclusion body pellet was solubilized in 50 mM Tris-HCl, 10 mM imidazole, 8M urea, pH 8. For purification of VEGFR1D2, solubilized protein was applied on Ni^{2+}-NTA resin (45 min, 20°C) in the presence of 300 mM NaCl. The His-tagged protein was refolded by equilibrating the resin in 50 mM Tris-HCl, 10 mM imidazole, 300 mM NaCl, pH 8 with decreasing concentrations of urea and then it was eluted with increasing imidazole concentration from 100 to 300 mM. The ^{15}N-labeled recombinant $VEGFR1D2_2$ was prepared according to Sambrook *et al.*[3] adding to the medium $^{15}NH_4Cl$ as the unique source of nitrogen. The expression, refolding, and protein purification were performed as described for the unlabeled protein. Refolded VEGFR1D2 was dialyzed overnight in 50 mM Tris-HCl, pH 7.0, and NaCl 250 mM at 4 °C.

The cleavage of the His-tagged protein (labeled and unlabeled) was performed in presence of Glutathione (3 mM reduced/ 0.3 mM oxidized) for 3 h at 30 °C, adding TEV protease to protein substrate in a molar ratio (protease:substrate) of 1:35. Finally, VEGFR1D2 was purified to homogeneity by size exclusion chromatography using a S75 column (GE Healthcare) equilibrated in 50 mM Tris-HCl and 150 mM NaCl, pH 7. Finally, it was concentrated until 0.9 mM by the Amicon Ultra system (10.000 MWCO, Millipore) and identified by LC-MS^2.

Table S1: Calculated and experimental molecular weight of synthesized peptides

**Peptide**	**MW_{Calc} (Da)**	**MW_{Exp} (Da)**
AcHx	1673.98	1673.32
AcHx-Thioester	1909.32	1909.2
AcHn	2000.03	2000.58
Hn0	2061.02	2062.04
Hn1	2118.05	2119.06
Hn2	2132.06	2133.92
Hn3	2146.13	2145.84
Hn4	2160.16	2161.11
Hn5	2174.18	2175.09
Hn6	2188.20	2189.14
EP0	3844.93	3844.97
EP1	3901.97	3904.01
EP2	3915.96	3916.03
EP3	3930.04	3930.03
EP4	3944.08	3944.03
EP5	3958.13	3958.06
EP6	3972.12	3972.08

Table S2: ^{1}H chemical shift assignment of the free EP6 peptide

Residue	δNH	δH$_α$	δH$_β$	δH$_γ$	δH$_δ$	δothers
**(CH3CO)**						
**E1**	8.32	4.31				
**K2**	8.23	4.33	1.76			
**F3**	8.56	4.62	3.18			7.22; 7.05; 7.18
**M4**	8.29	4,44	1.97; 1.88	2.45		
**D5**	8.11	4.67	2.78			
**V6**	8.41	4.11	2.01	0.80		
**Y7**	8.36	4.54	3.05; 2.93			7.12; 7.00
**Q8**	8.21	4.33	1.88	2.44; 2.38		7.11; 6.77
**L9**	8.37	4.31	1.56		0.79	
**K10**	8.33	4.28	1.75; 1.63	1.53		2.96
**Y11**	8.00	4.56	2.95; 2.84			7.04; 6.72
**K12**	8.12	4.35	1.65	1.40		2.92
**G13**	8.18	3.96				
**I14**	7.90	4.23	1.80	0.90		0.86
**C15**						
**Linker16**						
**Q17**	8.40	4.35	1.94; 1.90			
**I18**	8.19	4,22	1.72	1.07	0.76	
**M19**	8.57	4.55		3.13		
**R20**	8.39	4.39	1.70	1.52	3.13	
**I21**	8,04	4.28	1.49	1.10	0.73	
**K22**	7.92	4.36	1.95			
**P23**		4.43	2.20; 2.08	1.85		
**H24**	8.47	4.60	3.14; 3.06			7.11; 7.20
**Q25**	8.41	4.37	2.00; 1.91	2.20; 2.08		
**G26**	8.53	3.98				
**Q27**	8.25	4.41	1.75	2.25		
**H28**	8.65	4.66	3.18; 3.09			7.17; 7.26
**I29**	8.21	4.23	1.62	1.22; 0.90	0.67	
**G30**	8.55	3.99				
**E31**	8.20	4.32	2.01;2.21			
**T32**	8.01	4.34	4.02	1.11		
**S33**	8.11	4.50	3.90			
**(CONH2)**						

All chemical shifts are in parts/million and are relative to water protons (4.75 ppm)

Table S3: ^{1}H chemical shift assignment of the bound EP6 peptide

Residue	δNH	δH$_{\alpha}$	δH$_{\beta}$	δH$_{\gamma}$	δH$_{\delta}$	δothers
**(CH3CO)**						
**E1**	8.33	4.30				
**K2**	8.21	4.33	1.75			
**F3**	8.56	4.61	3.17			7.20; 7.05; 7.17
**M4**	8.30	4,43	1.95; 1.88	2.46		
**D5**	8.11	4.66	2.77			
**V6**	8.42	4.11	2.02	0.78		
**Y7**	8.36	4.52	3.05; 2.93			7.13; 6.97
**Q8**	8.22	4.32	1.89	2.45; 2.38		7.11; 6.78
**L9**	8.38	4.30	1.56		0.77	
**K10**	8.34	4.27	1.75; 1.63	1.53		2.96
**Y11**	8.01	4.55	2.96; 2.82			7.06; 6.75
**K12**	8.13	4.34	1.66	1.39		2.92
**G13**	8.18	3.95				
**I14**	7.90	4.24	1.79	0.91		0.86
**C15**	8.38	4.66				
**Linker16**						
**Q17**	8.41	4.35	1.93; 1.89			
**I18**	8.19	4,21	1.72	1.07	0.73	
**M19**	8.58	4.56		3.14		
**R20**	8.39	4.40	1.71	1.52	3.13	
**I21**	8,06	4.30	1.48	1.10	0.73	
**K22**	7.94	4.38	1.95			
**P23**		4.42	2.19; 2.08	1.83		
**H24**	8.47	4.60	3.14; 3.05			7.11; 7.18
**Q25**	8.41	4.36	2.00; 1.90	2.20; 2.09		
**G26**	8.53	3.98; 3.91				
**Q27**	8.25	4.42	1.75	2.25		
**H28**	8.65	4.66	3.18; 3.09			7.17; 7.26
**I29**	8.21	4.25	1.62	1.22; 0.90	0.67	
**G30**	8.55	4.00				
**E31**	8.20	4.33	2.01;2.21			
**T32**	8.01	4.34	4.02	1.11		
**S33**	8.11	4.49	3.90			
**(CONH2)**						

All chemical shifts are in parts/million and are relative to water protons (4.75 ppm)

Table S4: nonsequential NOEs involving backbone protons of EP6 peptide in the presence of VEGFR1D2.

Residue	Proton	Proton	Residue
**Tyr^{7}**	H_N	H_α	Met^{4}
**Gln^{8}**	H_N	H_α	Asp^{5}
**Leu^{9}**	H_N	H_α	Val^{6}
**Lys^{10}**	H_N	H_α	Tyr^{7}
**Tyr^{11}**	H_N	H_α	Gln^{0}
**Phe^{3}**	H_α	H_β	Val^{6}
**Met^{4}**	H_α	$H_{\beta2}/H_{\beta3}$	Tyr^{7}
**Val^{6}**	H_α	H_β	Leu^{9}
**Gln^{8}**	H_α	$H_{\beta2}/H_{\beta3}$	Tyr^{11}
**Phe^{3}**	H_N	H_N	Asp^{5}
**Met^{4}**	H_N	H_N	Val^{6}
**Met^{4}**	H_N	H_N	Tyr^{7}
**Asp^{5}**	H_N	H_N	Tyr^{7}
**Asp^{5}**	H_N	H_N	Gln^{8}
**Val^{6}**	H_N	H_N	Gln^{8}
**Gln^{8}**	H_N	H_N	Lys^{10}
**Leu^{9}**	H_N	H_N	Tyr^{11}
**Arg^{20}**	H_N	H_α	Ile^{29}
**His^{28}**	H_N	H_α	Ile^{21}
**Arg^{20}**	H_N	H_N	His^{28}
**Arg^{20}**	H_N	H_N	Gly^{30}
**His^{28}**	H_N	H_N	Gln^{25}
**Gly^{30}**	H_N	H_N	Ile^{18}

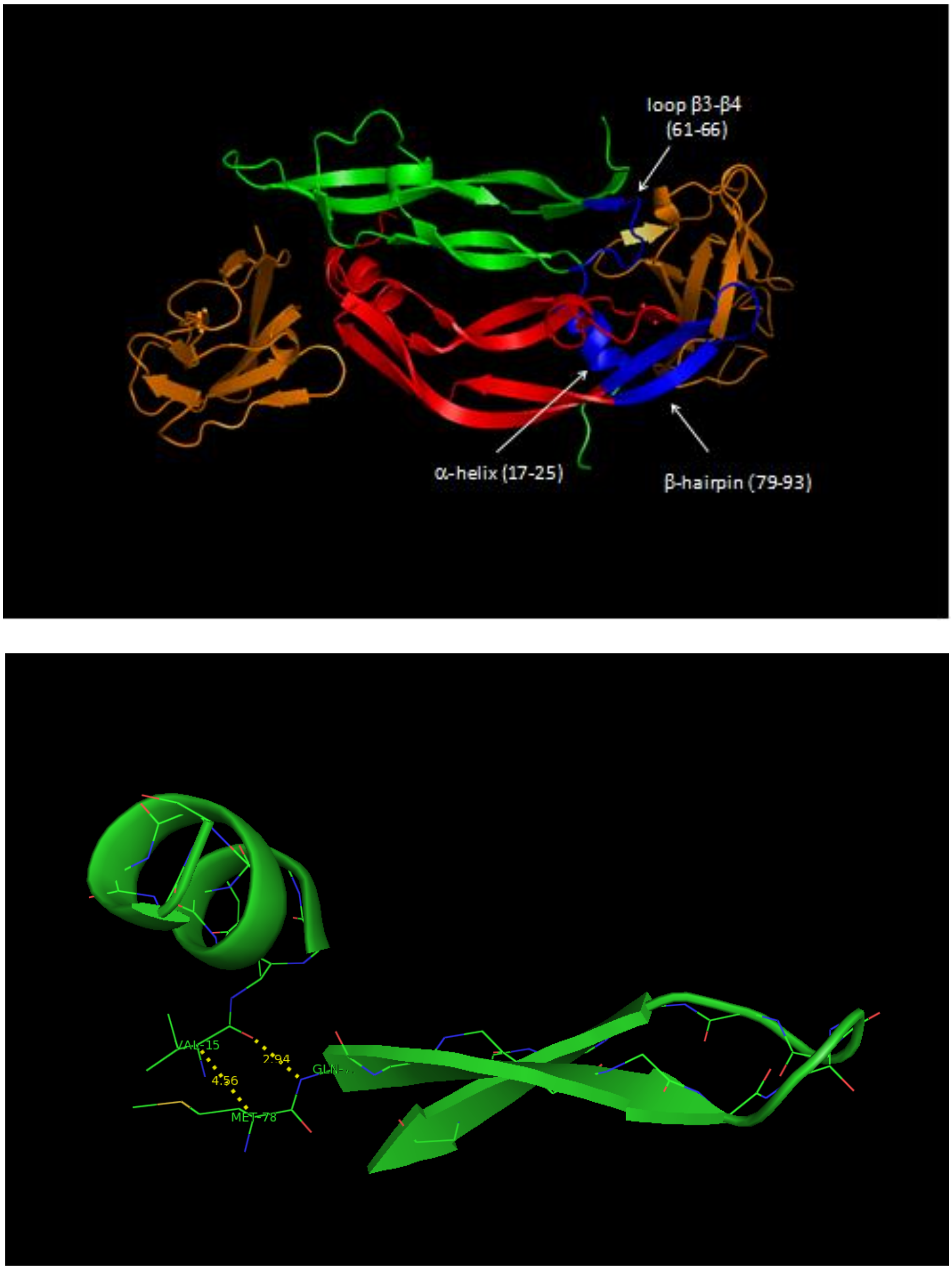

Figure S1: Molecular structure of the VEGF-VEGFR1D2 complex (C. Wiesmann, G. Fuh, H. W. Christinger, C. Eigenbrot, J. A. Wells and A. M. de Vos, *Cell* **1997**, *91*, 695-704). Top: General view of the VEGF-VEGFR1D2 complex. VEGF monomers are represented in green and red, whereas VEGFR1D2 is colored in orange. The interacting regions are highlighted in blue. Bottom: Molecular detail of VEGF structure highlighting the proximity between the α-helix (17-25) and the β-hairpin (79-93). Secondary structure elements are represented as green ribbon. The distances between C^{α} (Val15) – C^{α} (Met78) and O’ (Val15) – N^{α} (Gln79) are reported in yellow. This figure was made using PyMOL (The PyMOL Molecular Graphics System, Version 1.8 Schrödinger, LLC).

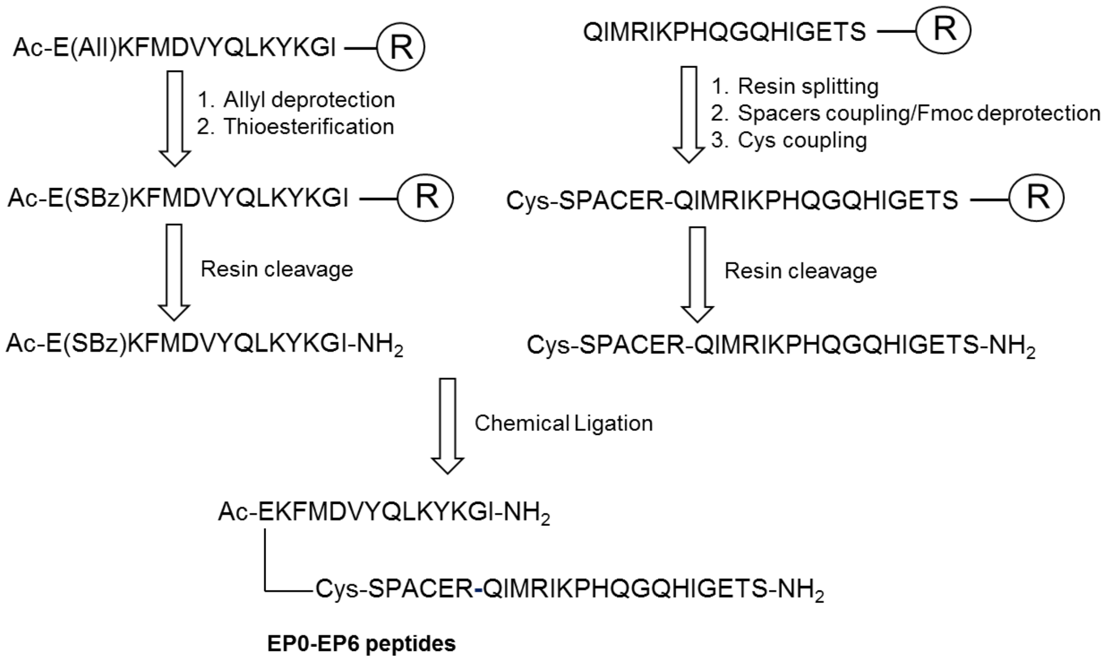

Figure S2. Schematic representation of the synthetic protocol for EPs peptides

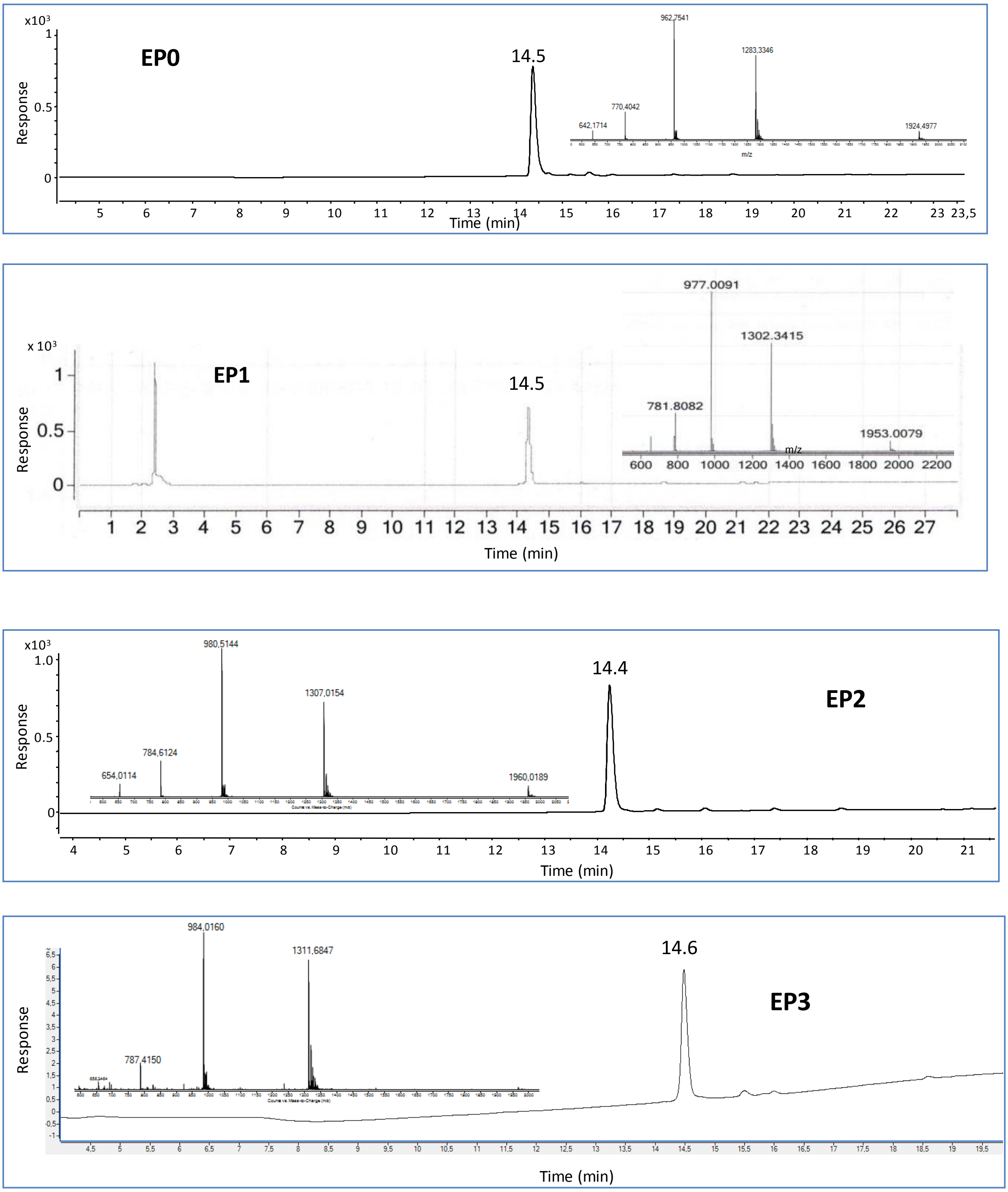
EP0
14.5
x10³
Response
Time (min)
962,7541
1283,3346
770,4042
642,1714
1924,4977
m/z
EP1
14.5
x 10³
Response
Time (min)
977.0091
1302.3415
781.8082
1953.0079
m/z
EP2
14.4
x10³
Response
Time (min)
980,5144
1307,0154
784,6124
654,0114
1960,0189
EP3
14.6
Response
Time (min)
984,0160
1311,6847
787,4150

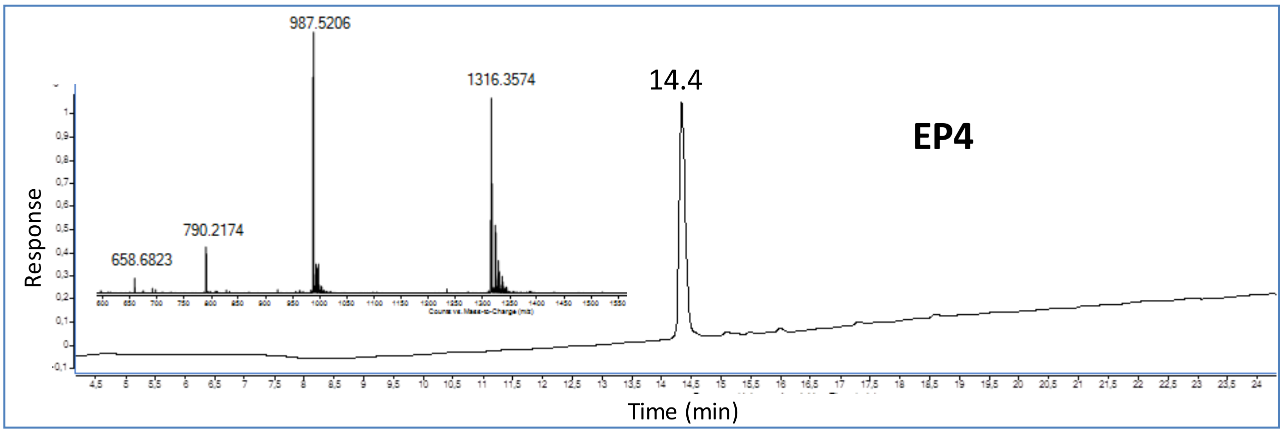

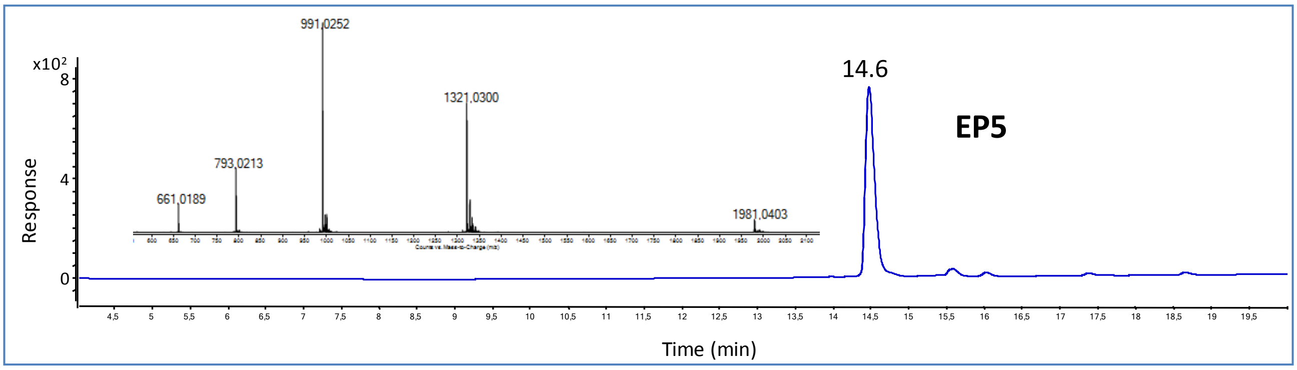

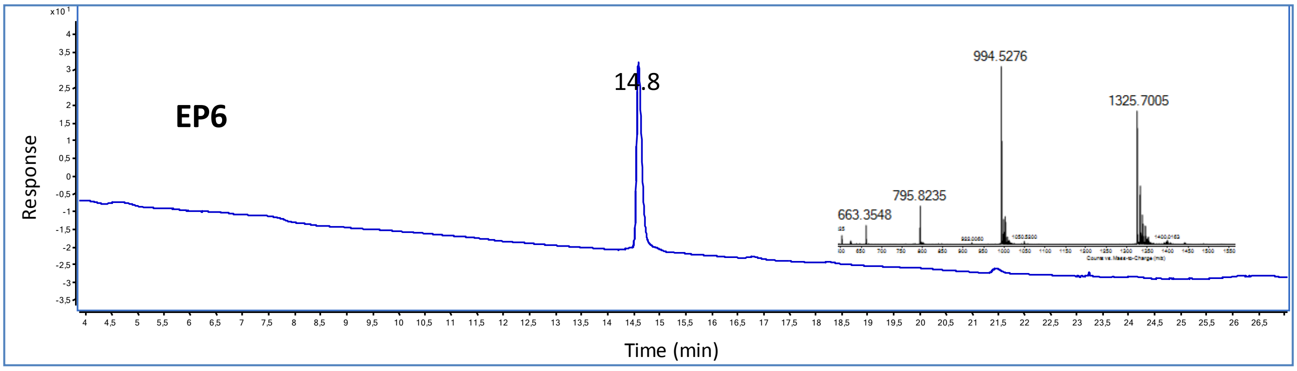

Figure S3. LC-MS analyses of purified EP0 – EP6 peptides. HPLC chromatograms were revealed at 210 nm. The mass spectrum of the indicated HPLC peak is shown in the inset.

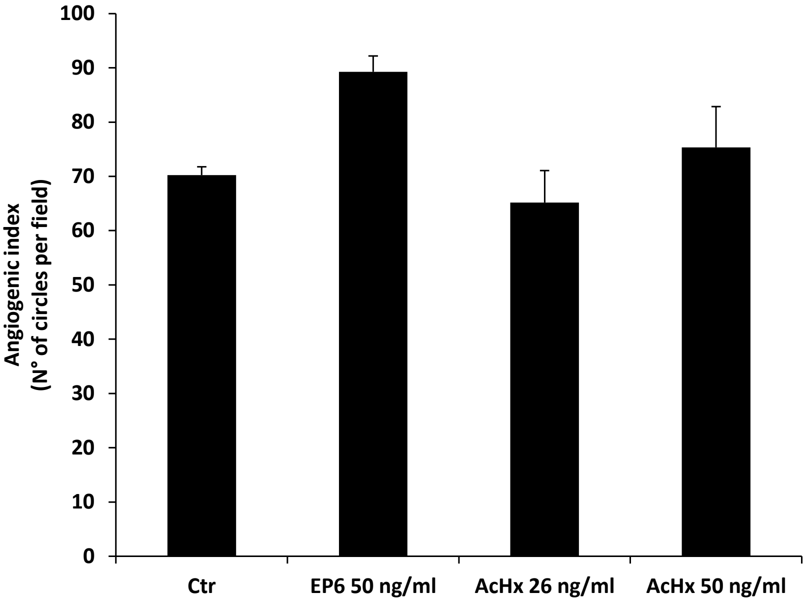

Figure S4. In vitro angiogenic properties of EP6 and AcHx peptides. HUVEC were plated onto a layer of basement membrane matrix (Matrigel) and incubated at 37 °C for 18 h in the presence of EP6 (50 ng/ml) and AcHx (50 or 26 ng/ml). After treatment, tubular structures and photomicrographs were quantified as angiogenic index, calculated as the number of complete circles counted/field by microscope image analysis (Bottom). *p<0.05 vs control.

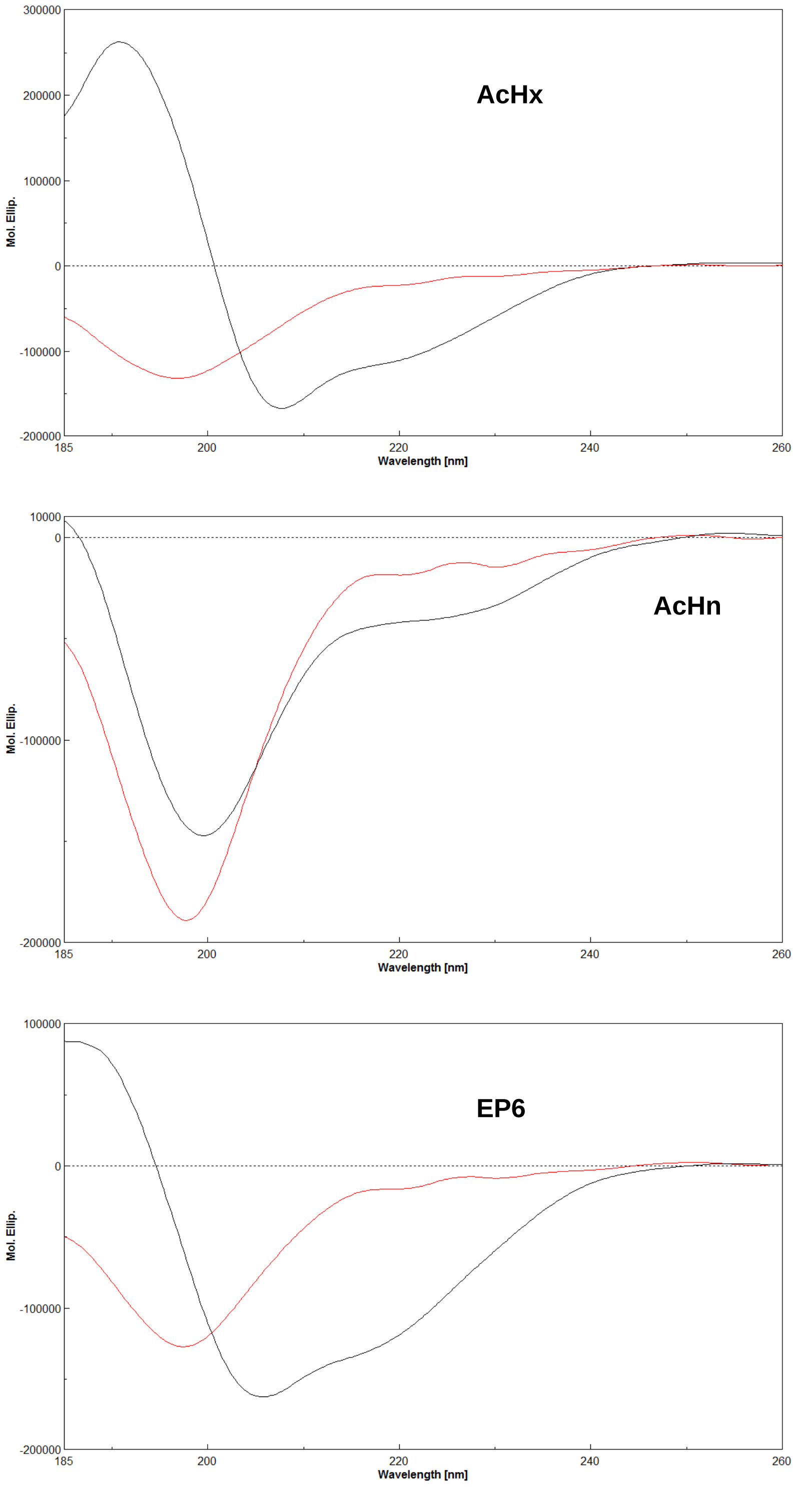

Figure S5. CD spectra of selected peptides. The CD spectrum in the absence (red) or in presence of 40% TFE (black) are reported.

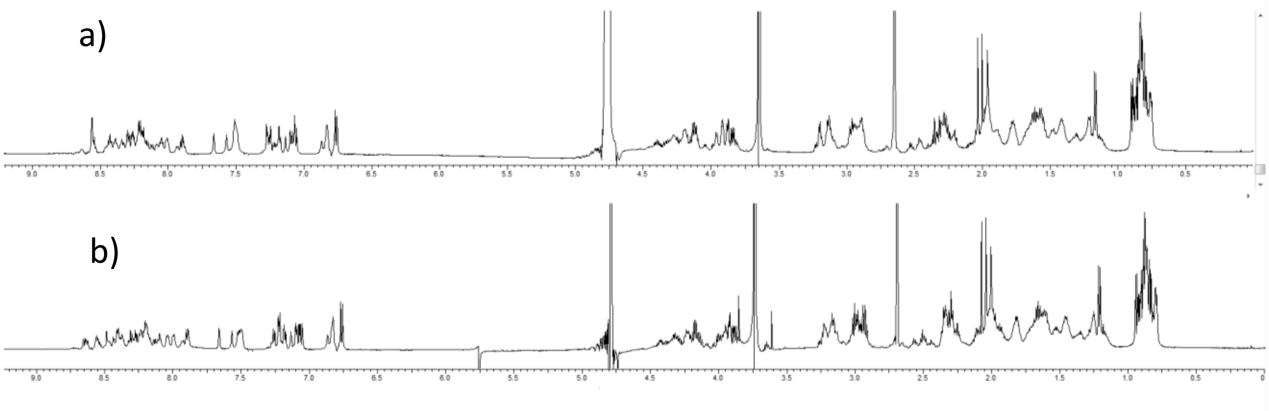

Figure S6. ^{1}H NMR spectra of (a) 0.55 mM EP6 in Tris-HCl 20 mM pH 7.5 and (b) in the presence of VEGFR1D2 (0.55 μM)

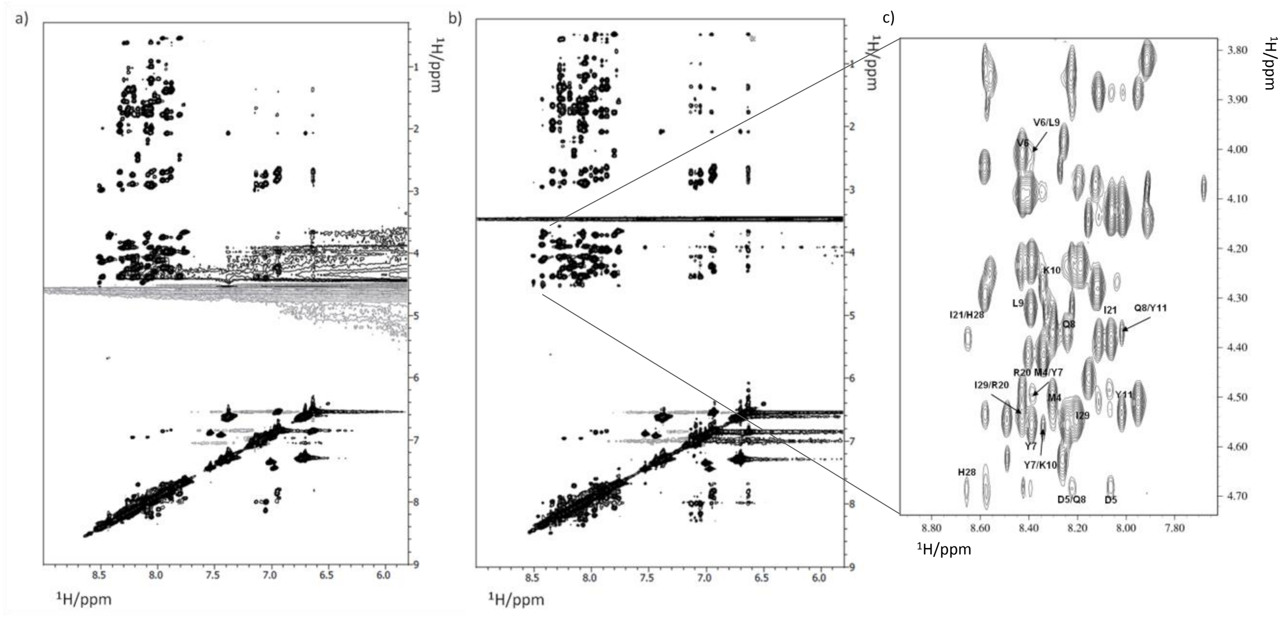

Figure S7: Sections of the 2D [^{1}H, ^{1}H] NOESY spectrum of free (a) and bound (b) EP6 peptide. c) Expanded region of αH-NH region of the 2D [^{1}H, ^{1}H] trNOESY spectrum.

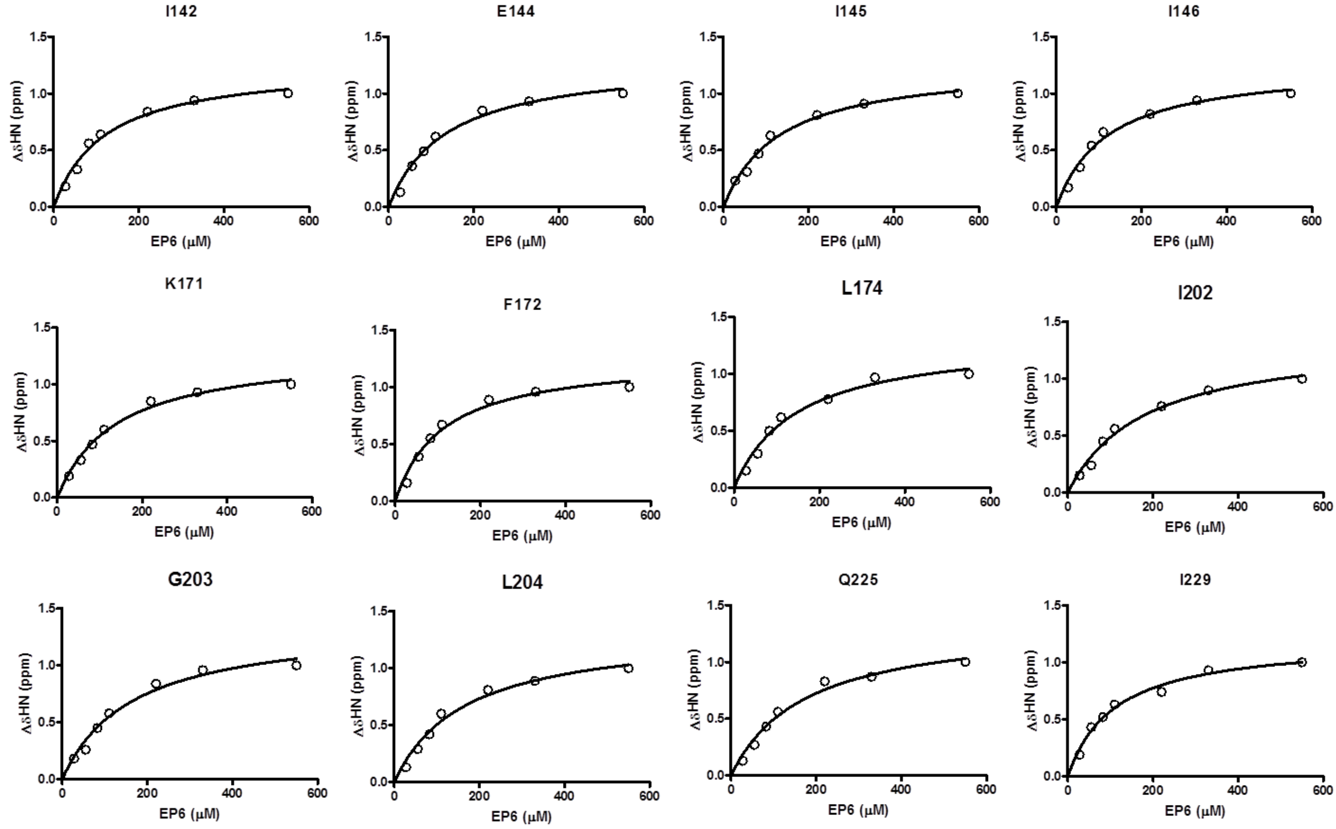

Figure S8: NMR binding studies of VEGFR1D2 with EP6. Chemical shift changes (ΔδHN) for the $H^{15}N$ resonances of the protein residues showing large variations in chemical shift upon formation of the EP6/VEGFR1D2 complex are reported as a function of the ligand concentration. The curves (solid line) represent the best fit solution of the equation that describes 1 : 1 complex formation. Data were fitted using GraphPad Prism.

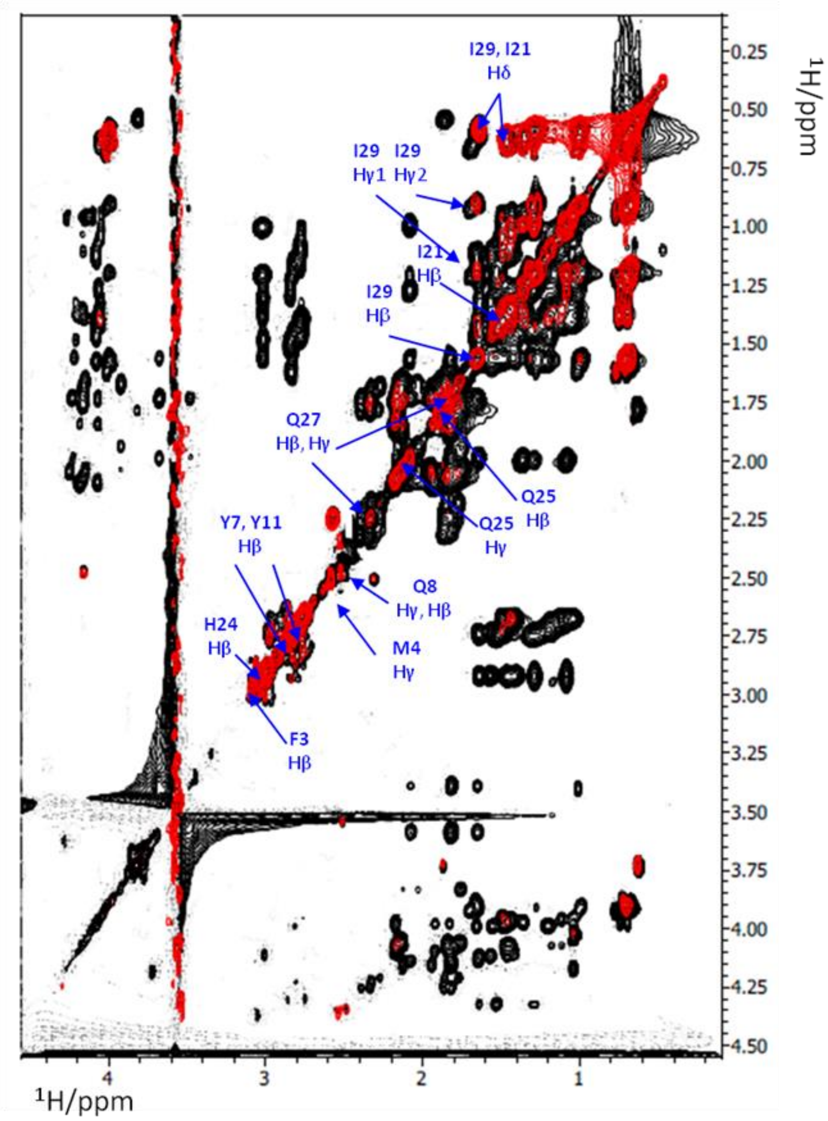

Figure S8: Aliphatic region of the reference 2D [^{1}H, ^{1}H] TOCSY spectrum (black) and 2D [^{1}H, ^{1}H] STD-TOCSY NMR spectrum (red) of 550 µM EP6 in the presence of 55 µM VEGFR1D2 (10:1). Residues showing the biggest STD effects are indicated.
